# Metagenomic Profiling of Antibiotic Resistance Genes and Mobile Genetic Elements in a Tannery Wastewater Treatment Plant

**DOI:** 10.1371/journal.pone.0076079

**Published:** 2013-10-01

**Authors:** Zhu Wang, Xu-Xiang Zhang, Kailong Huang, Yu Miao, Peng Shi, Bo Liu, Chao Long, Aimin Li

**Affiliations:** State Key Laboratory of Pollution Control and Resource Reuse, School of the Environment, Nanjing University, Nanjing, China; National University of Singapore, Singapore

## Abstract

Antibiotics are often used to prevent sickness and improve production in animal agriculture, and the residues in animal bodies may enter tannery wastewater during leather production. This study aimed to use Illumina high-throughput sequencing to investigate the occurrence, diversity and abundance of antibiotic resistance genes (ARGs) and mobile genetic elements (MGEs) in aerobic and anaerobic sludge of a full-scale tannery wastewater treatment plant (WWTP). Metagenomic analysis showed that *Proteobacteria*, *Firmicutes*, *Bacteroidetes* and *Actinobacteria* dominated in the WWTP, but the relative abundance of archaea in anaerobic sludge was higher than in aerobic sludge. Sequencing reads from aerobic and anaerobic sludge revealed differences in the abundance of functional genes between both microbial communities. Genes coding for antibiotic resistance were identified in both communities. BLAST analysis against Antibiotic Resistance Genes Database (ARDB) further revealed that aerobic and anaerobic sludge contained various ARGs with high abundance, among which sulfonamide resistance gene *sul1* had the highest abundance, occupying over 20% of the total ARGs reads. Tetracycline resistance genes (*tet*) were highly rich in the anaerobic sludge, among which *tet33* had the highest abundance, but was absent in aerobic sludge. Over 70 types of insertion sequences were detected in each sludge sample, and class 1 integrase genes were prevalent in the WWTP. The results highlighted prevalence of ARGs and MGEs in tannery WWTPs, which may deserve more public health concerns.

## Introduction

About 210,000 tons of antibiotics are produced annually in China, nearly half of which is used in animal agriculture for sickness prevention and production improvement [1,2]. The improper or illegal use of various antibiotics may result in the accumulation of residues in animal tissues including muscle, liver, kidney, skin and hair [3–5]. Leather production may facilitate the transfer of the antibiotic residues and resistant bacteria from animal tissues to the tannery wastewater. In addition, presence of various heavy metals [6] and biocides [7] in tannery wastewater contributes to co-selection of antibiotics and heavy metals in wastewater treatment plants (WWTPs) [8].

Current concerns focus on identification of heavy metal and antibiotic resistant bacteria isolated from tannery wastewater [9,10]. Previous studies have investigated the microbial community of activated sludge in tannery WWTPs through 16S rRNA gene amplification and sequencing [11,12]. However, information about abundance and diversity of antibiotic resistance genes (ARGs) in tannery WWTPs is limited. ARGs are often carried on mobile genetic elements (MGEs) including plasmids [13], transposons [14] and integrons [15], facilitating horizontal transfer among bacteria in WWTPs. Public health problems may arise from the ARGs spread and transfer in the environment.

Recently, metagenomic analysis combined with high-throughput sequencing has been considered as a promising culture-independent method of determining diversity and abundance of ARGs in various environments, such as activated sludge [13], drinking water [16], sediment [17] and soil [18]. This method has also shown great advantages on microbial community profiling due to its unprecedented sequencing depth, which has been used to characterize microbial community structure and function in activated sludge [19], buffalo rumen [20] and pipe biofilm [21].

This study aimed to use Illumina high-throughput sequencing to comprehensively investigate the microbial community structure and function of anaerobic and aerobic sludge in a full-scale tannery wastewater treatment plant, with emphasis on the abundance and diversity of ARGs and MGEs in the sludge.

## Materials and Methods

### Sludge sampling

Activated sludge samples were collected from the full-scale tannery WWTP of Boao Leather Industry Co., Ltd. geographically located in Xiangcheng City (Henan Province, China). We would like to state that the company has approved this study which did not involve endangered or protected species. Basically, a biological treatment system preceded by preliminary treatment including homogenization, chemical coagulation and primary settling was applied in this WWTP (Figure S1). The biological treatment system was composed of an up-flow anaerobic sludge reactor (UASB) and an integrated anoxic/oxic (A/O) reactor (Table S1). Anaerobic sludge was sampled from the UASB, and aerobic sludge was sampled from the last aerobic tank of the A/O reactor (Figure S1). The sludge samples were fixed with 50% ethanol (v/v) on site before transporting to laboratory for DNA extraction.

### DNA extraction

For DNA extraction, 4 ml of the anaerobic sludge and 10 ml of the aerobic sludge were separately centrifuged at 4,000 rpm for 10 min. Approximately 200 mg of pellet was recovered for total genomic DNA extraction in duplicate using FastDNA Soil Kit (MP Biomedicals, CA, USA) following the recommended protocol. The concentration and quality of the extracted DNA were determined with microspectrophotometry (NanoDrop® ND-1000, NanoDrop Technologies, Willmington, DE, USA).

### Illumina high-throughput sequencing and quality filtering

DNA samples (10 µg each) were sent to Beijing Genome Institute (Shenzhen, China) for high-throughput sequencing using Illumina Hiseq2000. A library consisting of about 180-bp DNA fragment sequences was constructed according to the manufacturer’s instructions before DNA sequencing. The strategy “Index 101 PE” (Paired End sequencing, 101-bp reads and 8-bp index sequence) was used for the Illumina sequencing, generating nearly equal amount of data for each sample. The metagenomic data were deposited in the publicly available database of MG-RAST (Meta Genome Rapid Annotation using Subsystem Technology) (http://metagenomics.nmpdr.org) under accession numbers 4494863.3 (anaerobic sludge) and 4494888.3 (aerobic sludge).

For quality control, the sequences contaminated by adapter or containing three or more unknown nucleotides were firstly removed using the quality control (QC) pipeline recommended by Beijing Genome Institute (Shenzhen, China). FASTX toolkit tools implemented in GALAXY [22] was then used to remove low quality sequences to ensure that more than 75% bases of each filtered read possessed Illumina quality greater than 30 (q30 indicating 0.1% sequencing error rate). The sequences containing one or more unknown nucleotides were removed by using a self-written Python script. The replicate sequences were removed by MG-RAST QC pipeline [23]. After the above quality filtering, a total of 9,194,933 and 8,652,320 quality-filtered reads were obtained for subsequent analysis of anaerobic and aerobic sludge metagenomes, respectively (Table S2).

### Combined taxonomic classification and function analysis

The quality-filtered reads were submitted to the MG-RAST (V3.3) for taxonomic classification and function analysis. Taxonomic analysis was conducted based on all the available annotation source databases in MG-RAST [19]. Both the phylogenetic information contained in the non-redundant database and the similarities to the rRNA databases were obtained for phylogenomic reconstruction of each sample. For functional assignments, the metagenomic data of anaerobic and aerobic sludge were annotated against SEED subsystems in MG-RAST at a cutoff of E-value < 10^-5^ [24]. The SEED established by Argonne National Lab (Argonne, USA) provides a suite of open source tools to enable researchers to create, collect, and maintain sets of gene annotations organized by groups of related biological and biochemical functions across many microorganisms [25]. A SEED subsystem is a collection of functional roles that together create a specific biological process or structural complex, which is created and curated by experts who specialize in an area relating to that subsystem [26]. The annotated sequences were sorted into 28 level 1 subsystems of SEED database to provide an overall profile of microbial functions. For the three level 1 subsystems of protein metabolism, stress response, and virulence, disease and defense, we further investigated specific variations of microbial functions at level 2. Additionally, the level 2 subsystem resistance to antibiotics and toxic compounds was further analyzed at level 3.

### ARGs and MGEs analysis

A local database of resistances genes was created by downloading all sequences from Antibiotic Resistance Database (ARDB) (23,137 sequences of 380 ARGs encoding resistance to 249 antibiotics) [27]. All quality-filtered reads were compared against the collection of ARGs using BLAST (BLASTx) at a cutoff of E-value <10^-5^. A read was annotated as an ARG according to its best BLAST hit if the hit had a sequence similarity of above 90% over an alignment of at least 25 amino acids [13,16,17]. Local databases of insertion sequences (ISs) and integron integrase genes were separately created by downloading ISs sequences from the ISfinder (2,578 sequences, 22 families of insertion sequences) [28] and integrase genes from the INTEGRALL (1,447 sequences) [29]. A read was identified as an insertion sequence or integrase gene if the BLAST hit (BLASTn with the E-value cut-off at 10^-5^) had a nucleotide sequence identity of above 90% over an alignment of at least 50 bp [13,17].

## Results and Discussion

### Taxonomic analysis of microbial communities

**Figure 1:**
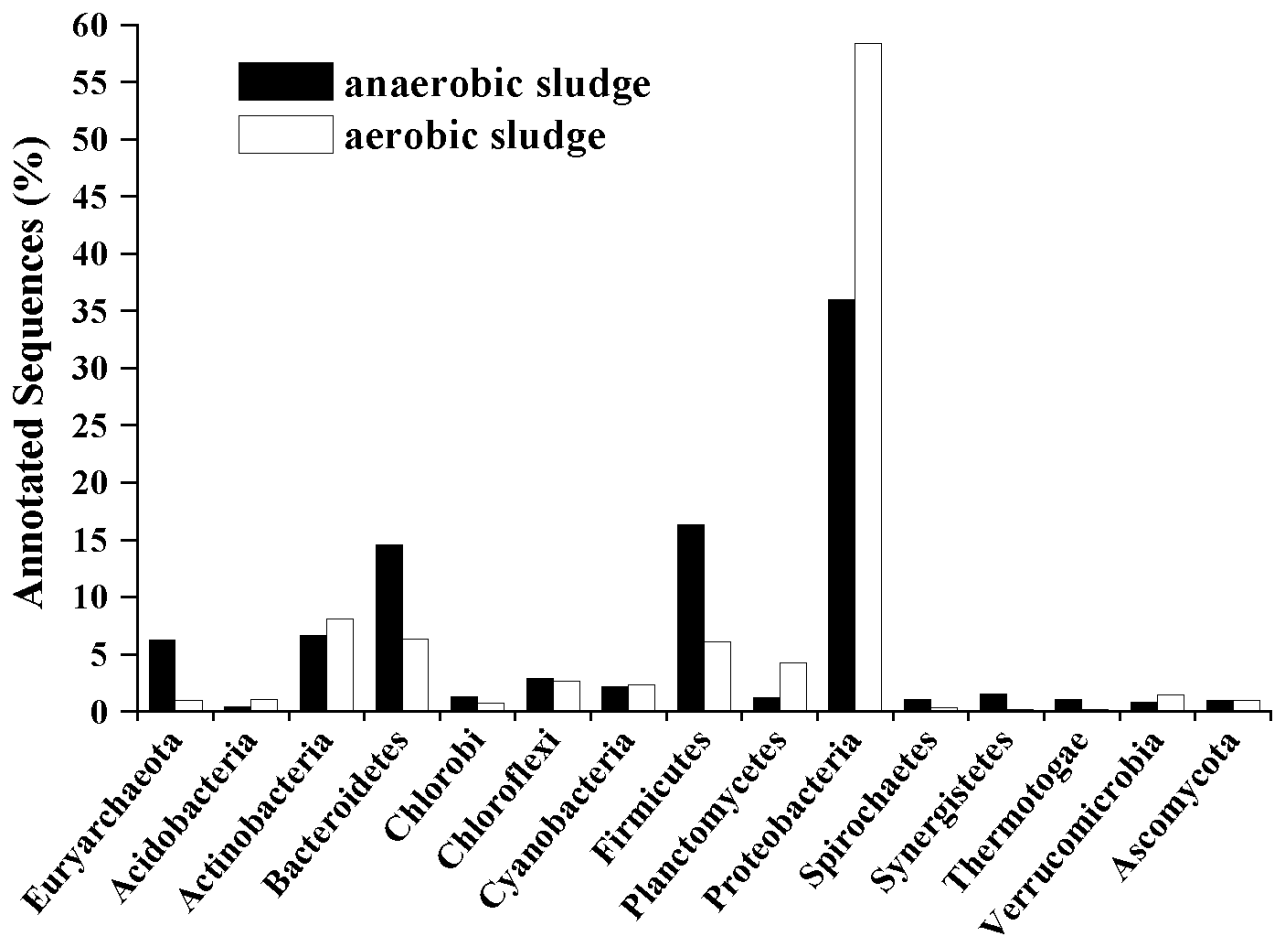
Combined taxonomic phylum information of anaerobic and aerobic sludge. The phyla shown have relative abundance over 1% of total sequencing reads annotated at phylum level in either anaerobic or aerobic sludge.

Taxonomic affiliation of both predicted proteins and rRNA genes sequences in the sludge were conducted based on all the available annotation source databases in MG-RAST. Bacteria were predominant in both sludge samples, occupying 88.41% and 93.79% of all annotated sequences in the anaerobic and aerobic sludge, respectively (Figure S2). *Proteobacteria* (35.95% and 58.36% of annotated reads from the anaerobic and aerobic sludge, respectively), *Firmicutes* (16.31% and 6.08%, respectively), *Bacteroidetes* (14.53% and 6.36%, respectively) and *Actinobacteria* (6.66% and 8.06%, respectively) were the dominant phyla in the anaerobic and aerobic sludge (Figure 1). This result is supported by a previous study indicating that *Proteobacteria* was the most dominant phylum in sewage sludge, followed by *Bacteroidetes*, *Firmicutes* and *Actinobacteria* [30]. Microarray [31] and DNA cloning [32] have also shown that *Proteobacteria* often dominate in activated sludge. Proteins and carbohydrates often have high concentration in tannery wastewater [33], and *Bacteroidetes* are well known degraders of the organic matters due to the presence of numerous genes encoding protein or carbohydrate degrading enzymes in their genomes [34]. The genomes of *Bacteroidetes* are highly plastic and frequently reorganized, so they can adapt to and dominate in different ecological niches, e.g. soil, ocean, freshwater and activated sludge [30,34].

Oxygen concentration is an important factor shaping microbial community structures in WWTPs, and may make huge contributions to the observed divergence of microbial community structure between anaerobic sludge and aerobic sludge. Our results demonstrated that the phyla of *Synergistetes* and *Thermotogae* (known to be anaerobic bacteria) had higher abundance in the anaerobic sludge than in the aerobic sludge (Figure 1). Lefebvre et al. [11] also indicated that *Synergistetes* occupied 4% of total bacteria population in a UASB treating tannery wastewater, but the phylum was absent in aerobic sludge. *Synergistetes* [35] can use amino acids from the breakdown of proteins and peptides by other organisms, which in return provides short-chain fatty acids and sulfate for terminal degraders including methanogens and sulfate-reducing bacteria. At genus level, aerobic bacteria *Burkholderia* and *Pseudomonas* were predominant in the aerobic sludge (Table S3). The anaerobes *Bacteroides* , *Clostridium* and *Desulfovibrio* dominated in the anaerobic sludge, but they had relatively low abundance in the aerobic sludge (Table S3). As the strictly anaerobic Gram-positive hydrogen–producing bacteria, the genus *Clostridia* was most dominant within the phylum *Firmicutes* in the anaerobic sludge. This may result from the capability of *Clostridia* to form endospores to survive under unfavorable environments [36]. It is not surprising that sulfate-reducing bacteria (e.g. *Desulfovibrio* ) had high abundance in anaerobic sludge, since sulfate is one of the common pollutants in tannery wastewater [33].

The relative abundance of Archaea in the anaerobic sludge was about three times higher than that of the aerobic sludge. In anaerobic digestors, oxygen unavailability and gentle physical disturbance might contribute to archaeal survival [37,38]. Among Archaea, *Euryarchaeota* had the highest abundance in the anaerobic sludge. Previous studies have confirmed that *Euryarchaeota* dominates in anaerobic sludge by using 16S rRNA gene library analysis [39] and 454-pyrosequencing [40]. This study showed that eukaryotes had nearly equal abundance in the two samples (Figure S2) and the contents of known viruses and other unclassified organisms occupied negligible proportions (<0.28% each) (Figure S2). *Ascomycota*, the largest phylum of Fungi [41], was the most dominant eukaryote in both anaerobic (1.00%) and aerobic sludge (0.99%) (Figure 1).

### Functional analysis of microbial communities

Functional analysis was also conducted by using MG-RAST program in the present study. A total of 843,224 (9.17%) sequences of the anaerobic sludge and 600,482 (6.94%) sequences of the aerobic sludge could be annotated against SEED level 1 subsystems database. The annotation proportions were higher than the percentage of successfully assigned sequences (3.03%) reported by Yu and Zhang [19] using Illumina sequencing technology to characterize the structure and function of a sewage sludge community. However, previous studies have reported that about 25% of the Illumina reads and over 36% of the pyrosequencing reads from soil metagenomes had a significant match in the SEED database [42]. Thus, the divergences of annotation proportions may result from the differences in environmental sample types and microbial communities.

**Figure 2:**
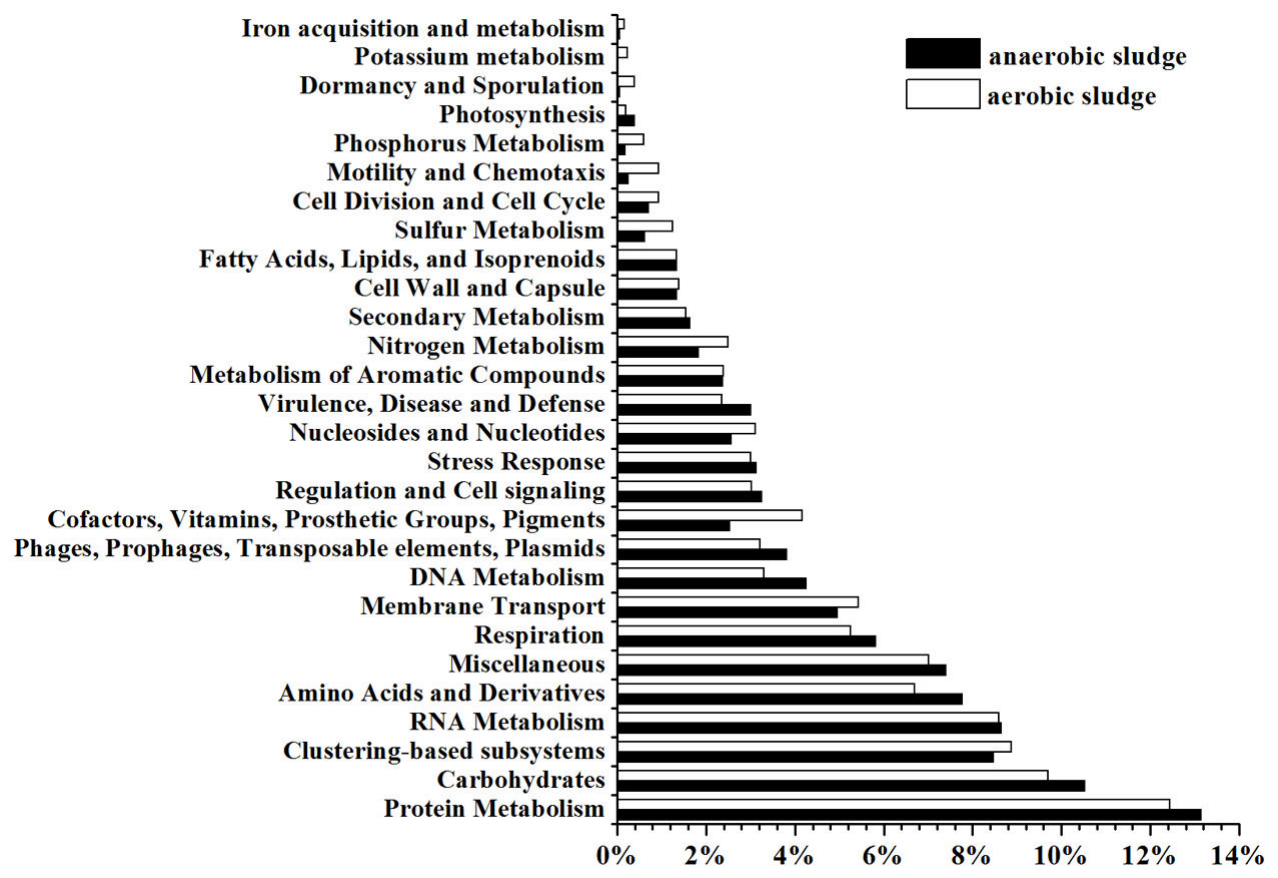
Relative distribution of sequencing reads in major level 1 subsystems in anaerobic and aerobic sludge. Metagenomic data were annotated against SEED subsystems in MG-RAST at a cutoff of E-value < 10^-5^.

Figure 2 shows the relative distribution of 28 basic metabolic categories within the anaerobic and aerobic sludge metagenomes. Protein metabolism was the most abundant category in the anaerobic and aerobic sludge, which is similar to the findings obtained from sewage sludge [19]. However, among the metabolic categories, the category of carbohydrates often has the highest abundance in soil metagenomes [42,43].

Protein metabolism, the most abundant category in both the samples, was selected for further analysis using the MG-RAST program. The annotated sequences of protein metabolism in anaerobic sludge were assigned to five subsystems at level 2, among which protein biosynthesis was the most abundant subsystem (56.87% of annotated sequences in protein metabolism), followed by protein folding (19.01%) and protein degradation (15.58%) (Figure S3A). However, protein degradation had higher abundance than protein folding in aerobic sludge. In aerobic sludge, microorganisms use molecular oxygen (O_2_) for respiration or oxidation of nutrients to obtain energy, and inevitably generate reactive oxygen species, such as hydrogen peroxide (H_2_O_2_) and highly reactive hydroxyl radicals (·OH) able to induce oxidative damage to proteins in microorganisms [44]. Microorganisms have to remove oxidized proteins through protein degradation since accumulation of such damaged proteins can cause cellular and organismic dysfunction [44,45]. Additionally, the protein degradation may contribute to energy production in the aerobic sludge where the available organic carbon is relatively insufficient in comparison with under anaerobic environment (Table S1) [46].

Figure S3B shows the relative distribution of level 2 subsystems in level 1 category of stress response. Oxidative stress, heat shock, detoxification and osmotic stress were the four most abundant subsystems in anaerobic and aerobic sludge, which might result from the high levels of various toxic chemicals and salts in the extreme environment of tannery wastewater [33]. However, acid stress subsystem was richer in the aerobic sludge than in the anaerobic sludge (Figure S3B). Oxygen availability may facilitate conversion of ammonia to nitrite or nitrate to induce pH decrease [47,48], which might result in the higher level of acid stress in aerobic sludge. Anaerobic sludge had higher abundance of dimethylarginine metabolism subsystems than aerobic sludge (Figure S3B). The aerobic sludge contained high level of nitrate nitrogen (Table S1), and it is known that nitric acid is able to inhibit arginase activity [49].

Figure S3C shows the relative distribution of level 2 subsystems in virulence, disease and defense of anaerobic and aerobic sludge. The genes involved in virulence, disease and defense occupied 3.01% and 2.35% of the total reads annotated by SEED subsystems in the anaerobic and aerobic sludge, respectively. This is generally consistent with abundance of the genes in sewage sludge [19]. Resistance to antibiotics and toxic compounds, an extremely important feature for microbial survival and adaptation in contaminated environments [50], was the most abundant subsystem in both the samples, occupying over 60% of the annotated sequences in the category of virulence, disease and defense in each sample. To better understand antibiotic resistance in the sludge, the subsystem of resistance to antibiotics and toxic compounds was further analyzed at level 3 (Table S4). Both the two samples showed presence of genes conferring resistance to antibiotics (e.g. fluoroquinolones and aminoglycosides) and heavy metals (e.g. copper and arsenic). Fluoroquinolones are widely used as animal feeding additive [51], and fluoroquinolone resistance genes have often been detected in animal breeding farms [52]. Generally, tannery wastewater is characterized with high concentrations of heavy metals [6], and the co-selection of antibiotics and heavy metals may contribute to the prevalence of antibiotic and heavy metal resistance genes in the sludge [8].

### Abundances and diversity of ARGs

**Figure 3:**
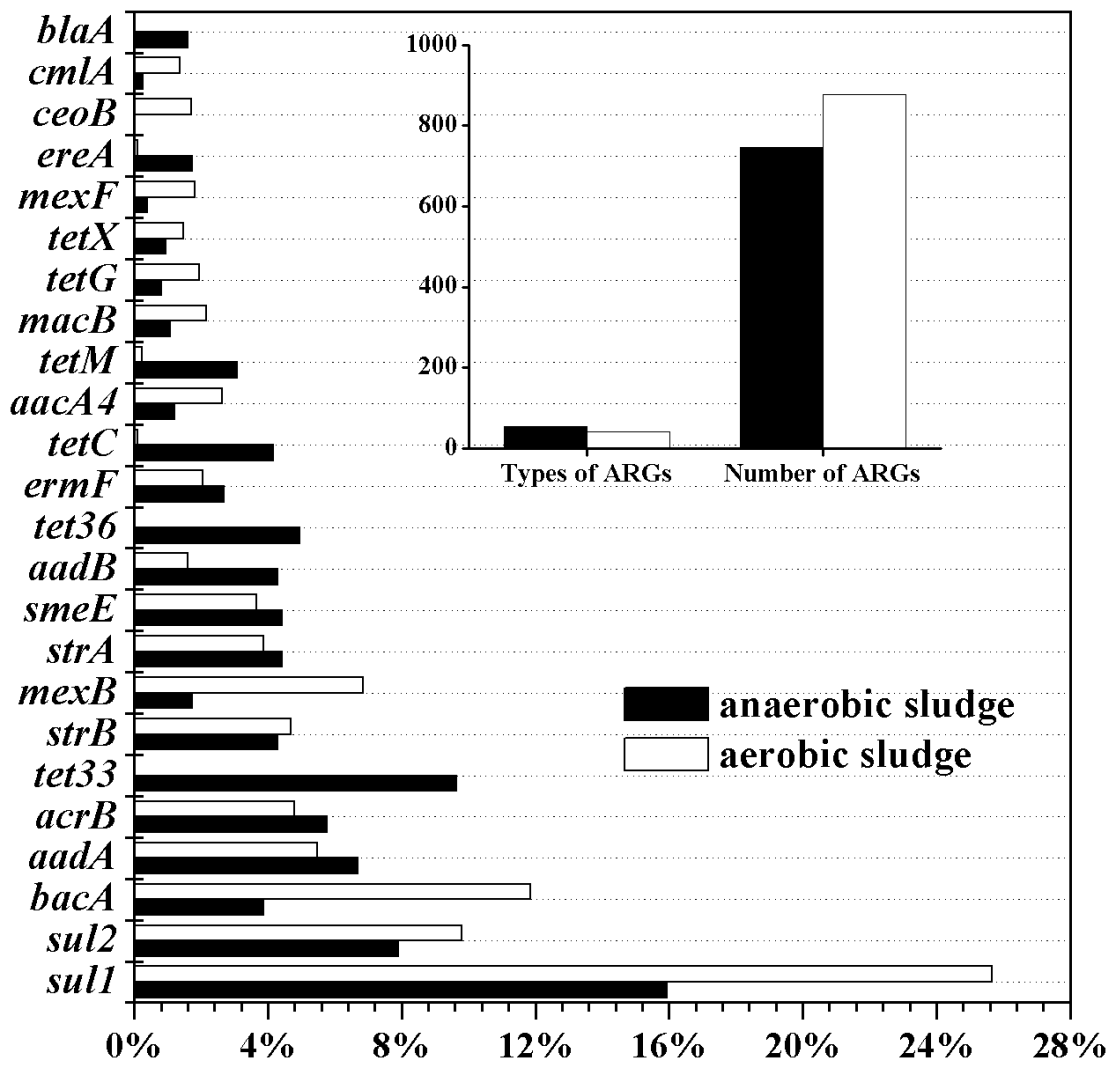
Types and relative abundance of antibiotic resistance genes (ARGs) in anaerobic sludge and aerobic sludge. The ARGs shown have relative abundance over 1% of the total ARGs reads in either anaerobic or aerobic sludge.

In order to comprehensively explore the ARGs present in the tannery WWTPs, we compared all the high-throughput sequencing reads against the ARDB protein database. BLAST analysis showed that a total of 747 reads (0.0081%) of the anaerobic sludge and 877 reads (0.0101%) of the aerobic sludge were assigned to 54 and 42 types of the known ARGs, respectively (Figure 3, Table S5). A total of 48 kinds of multidrug transporters that pump a broad spectrum of antibiotics out of cells were also included in the ARDB database. Due to their contribution to antibiotic resistance phenotype, multidrug transporters have the similar functions of ARGs and are often considered in antibiotic resistance analysis [13,16,17]. In this study, 109 reads (10 types) from anaerobic sludge and 206 reads (12 types) from aerobic sludge were annotated as multidrug transporters.

The proportions of the total ARGs identified in this study were comparable to the results previously obtained from sewage sludge metagenome by Illumina high-throughput sequencing (0.007%) [13] and sewage effluent metagenome by 454-pyrosequencing (0.012%) [53]. However, the annotation proportions of this study were lower than those of antibiotic contaminated sediments (0.02%–1.71%) [17], and higher than those of drinking water (0.0004-0.0071%) [16] and marine water (0.0017%) [53]. Previous studies have shown that sewage treatment plants serve as important reservoirs of environmental ARGs [54,55], and this study reveals that tannery WWTPs can also be considered as the sources of environmental ARGs. The wide use of antibiotics for animal health protection and growth stimulation contributes the prevalence of ARGs in tannery WWTPs [54].

**Figure 4:**
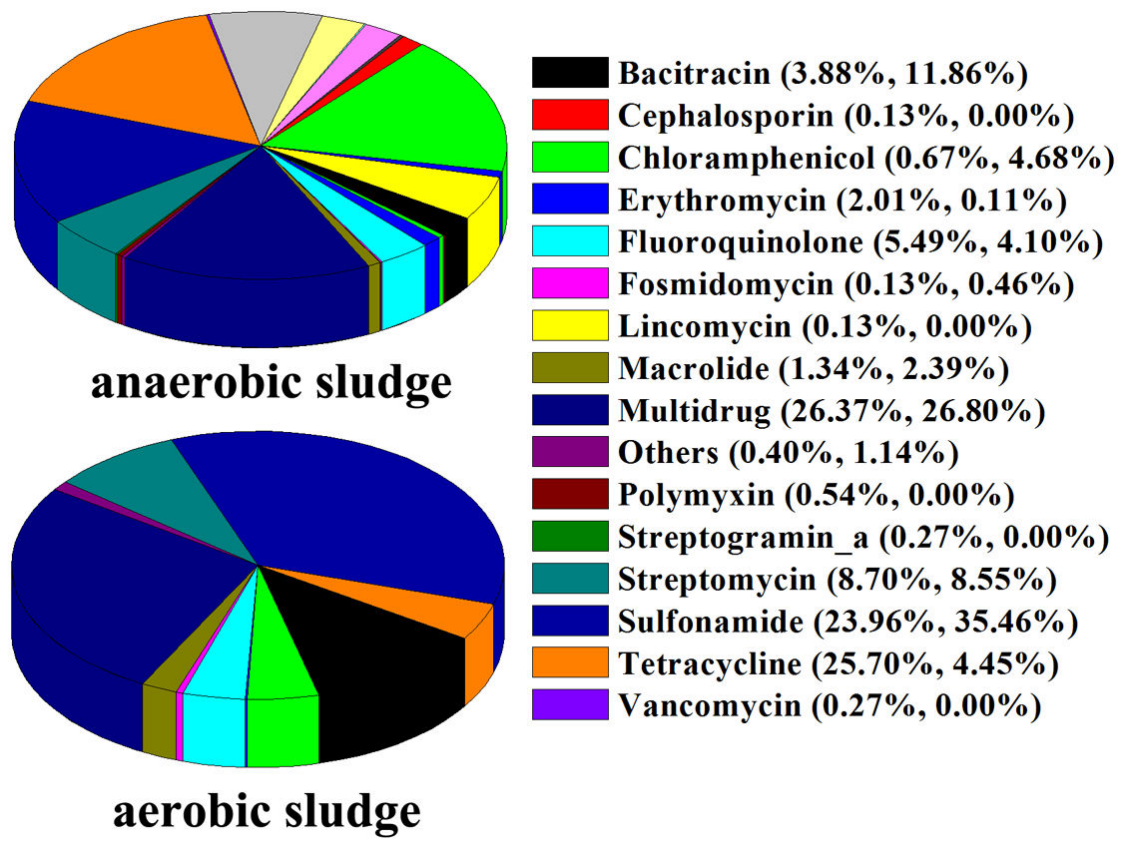
Antibiotic resistance gene patterns in anaerobic and aerobic sludge. The resistance genes were grouped after alignment of the high-throughput sequencing reads against ARDB database. The two percentages shown in the brackets represents the proportions of the reads of each antibiotic resistance gene (ARG) in the total reads of all the identified ARGs in anaerobic (first number) or aerobic (last number) sludge.

Our results demonstrated that the multidrug resistance genes, tetracycline resistance genes (*tet*) and sulfonamide resistances genes (*sul*) were common in the anaerobic sludge, each occupying over 20% of the reads involved in antibiotic resistance (Figure 4). However, in the aerobic sludge, *sul* genes had the highest abundance (35.46% of the total ARGs reads), followed by the multidrug resistance genes (26.80%) and bacitracin resistance genes (11.86%) (Figure 4). The prevalence of *tet* and *sul* genes in the two activated sludge samples may result from the frequent use of tetracycline and sulfonamides for livestock purposes in China [56,57].

Among the identified ARGs, the dihydropteroate synthase gene *sul1* that confer resistance to sulfonamides had the highest abundance in both the anaerobic and aerobic sludge (Figure 3). Besides *sul1*, sulfonamide resistance gene *sul2* also showed high levels, occupying 7.90% and 9.81% of total ARGs reads in the anaerobic and aerobic sludge, respectively (Figure 3). Sulfonamides with high solubility can persist in the environment [1,58], resulting in the high abundance of *sul1* and *sul2* in the tannery WWTP. *Tet* genes were highly rich in the anaerobic sludge, occupying 25.70% of total ARGs reads, but only 4.45% were annotated as *tet* genes in aerobic sludge (Figure 4). Among the *tet* genes, *tet33* had the highest abundance (72 reads, 9.64% of the total ARGs’ reads) in the anaerobic sludge, while the gene was absent in the aerobic sludge. Similarly, *tetC*, *tet36* and *tetM* were common in the anaerobic sludge, but they had lower abundance or were absent in the aerobic sludge (Figure S4). However, *tetC* often had higher levels than other *tet* genes in the aerobic tank of sewage treatment plants [55]. Tetracycline is not biodegradable and can be easily adsorbed to sludge [59]. In this study, the UASB was run under long sludge retention time with high biomass (Table S1), which may facilitate adsorption of tetracycline to sludge, subsequently resulting in higher abundance of the *tet* genes in the anaerobic sludge than in the aerobic sludge.

It should be noted that the results of ARGs abundance and diversity obtained by ARDB alignment were different from those derived from MG-RAST analysis. The divergence may result from the difference in reference databases and alignment methods. The BLAST program was used for ARDB-based analysis in this study, but MG-RAST relies on BLAT for similarity search, which is less sensitive than BLAST [60]. ARDB unified most of the publicly available ARGs and is considered as a comprehensive and higher-coverage annotation source for ARGs analysis [13,17]. However, the subsystem of “Resistance to antibiotics and toxic compounds” within SEED database contains incomplete information of ARGs (http://theseed.uchicago.edu/FIG/subsys.cgi).

### Abundances and diversity of MGEs

The mobility and acquisition of ARGs depends on MGEs, such as plasmids, transposons, ISs and integrons. In this study we focused our analysis on ISs and integrons. Search in INTEGRALL database showed that a total of 130 reads (0.0014%) of the anaerobic sludge and 327 reads (0.0038%) of the aerobic sludge could be assigned to integrase genes (Table S6). The most abundant integrase gene was *intI1*, which occupied 80.00% and 76.45% of alignment hits of anaerobic sludge and aerobic sludge, respectively. Previous studies have also shown the prevalence of integrons in WWTPs [61], including class 1 integrons carrying various ARGs in both aerobic and anaerobic sewage sludges [54,62]. It was found that 2 sequencing reads from the aerobic sludge could be annotated as *IntINeu*, a chromosomal integron integrase gene from *Nitrosomonas europaea*, which has been shown to be able to excise and integrate several resistance gene cassettes [63]. This is consistent with our results that the genera *Nitrosomonas* had higher abundance in aerobic sludge than in anaerobic sludge (Table S3).

Alignment against the ISfinder database demonstrated that a total of 586 reads (0.0064%) of the anaerobic sludge and 687 reads (0.0079%) of the aerobic sludge could match 76 and 81 types of known ISs, respectively. However, the two samples shared only 29 common types (Table S7). Among the ISs in the anaerobic sludge, IS*Efa4* (133 reads, 22.70%) had the highest abundance, followed by IS*Ecp1* (129 reads, 22.01%) and IS*Dde1* (99 reads, 16.89%), but they had much lower abundance or were absent in the aerobic sludge. IS*Dde1* is usually located in the cells of strictly anaerobic sulfate-reducing bacteria *Desulfovibrio* *desulfuricans* [64], and IS*Efa4* is often carried by gut pathogen *Enterococcus faecium* [65]. This is consistent with our results that the genera *Desulfovibrio* and *Enterococcus* had higher abundance in anaerobic sludge than in aerobic sludge (Table S3). Different from the anaerobic sludge, the aerobic sludge was dominated by IS*Vsa3* (106 reads, 15.43%), IS*Sm2* (70 reads, 10.19%) and IS*Pps1* (49 reads, 7.13%) (Table S7). IS*Pps1* prevalent in activated sludge [13] and drinking water [16] has a very broad host range including Gram-negative (*Alpha*-, *Beta*-, and *Gamma-Proteobacteria*) and Gram-positive bacteria ( *Arthrobacter* *aurescens* TC1) [66].

Our results suggested that integrons and ISs prevalent in the two sludge samples might play important roles in acquisition and mobility of various ARGs among the bacterial species. Therefore, the discharge of the tannery wastewater into the environments may be of great public health concern, since the MGEs in surface water and groundwater could potentially transfer antibiotic resistance to the bacteria in drinking water or food chain [67].

In conclusion, this study demonstrated that high-throughput sequencing provided a comprehensive insight in microbial community structures and functions of the aerobic and anaerobic sludge in tannery WWTPs. Metagenomic analysis revealed prevalence of a variety of ARGs in tannery WWTPs. Sulfonamide resistance genes had high abundance in both the sludge samples, but tetracycline resistance genes preferred anaerobic environment. Various MGEs including integrons and ISs were prevalent in the tannery WWTP.

## Supporting Information

Figure S1 **Operational processes of the full-scale tannery wastewater treatment plant.** A: Influent; B: Adjusting tank; C: Flocculant dosing system; D: Primary sedimentation Tank; E: Up-flow anaerobic sludge reactor; F: Integrated A/O reactor; G: Secondary sedimentation tank; H: Effluent; ①: Influent of up-flow anaerobic sludge reactor; ②: Effluent of up-flow anaerobic sludge reactor; ③: Effluent of integrated A/O reactor. The anaerobic and aerobic sludge samples were collected from Site E and the last aerobic tank of Site F, respectively. The water samples were collected from Sites ①, ② and ③ for water quality analysis.
(DOCX)

Figure S2 **Combined taxonomic domain of anaerobic and aerobic sludge.** Each sequencing read is assigned to bacteria, eukaryota, archaea, viruses, and other sequences.
(DOCX)

Figure S3 **Functional analysis of the microbial community in anaerobic and aerobic sludge by using MG-RAST annotation.** This figure shows the relative distribution of level 2 categories in level 1 categories of protein metabolism (A), stress response (B), and virulence, disease and defense (C).
(DOCX)

Figure S4 **Relative abundance and of different tetracycline resistance genes (*tet*) in anaerobic and aerobic sludge.**
(DOCX)

Table S1 **Operational conditions of the tannery wastewater treatment plants.**
(DOCX)

Table S2 **Information of sequence quality control for the metagenomic data of the sludge samples.**
(DOCX)

Table S3 **Abundance of different genera in anaerobic and aerobic sludge.** The abundance is presented in terms of percentage in total classified sequences in a sample. Representative taxa with abundance of over 0.25% in either anaerobic or aerobic sludge are shown (Sorted alphabetically by domain, phylum and then genus).
(DOCX)

Table S4 **Relative distribution of level 3 categories in level 2 category of “resistance to antibiotics and toxic compounds” based on MG-RASR analysis.**
(DOCX)

Table S5 **Matched high-throughput sequencing reads of ARGs in anaerobic and aerobic sludge against ARDB.** (Ranked by sequencing number of the identified ARGs in anaerobic sludge).
(DOCX)

Table S6 **Matched high-throughput sequencing reads of integron integrase genes in anaerobic and aerobic sludge against the INTEGRALL database.** (Ranked by reads number of the identified integron integrase genes in aerobic sludge).
(DOCX)

Table S7 **Matched high-throughput sequencing reads of insertion sequences in anaerobic and aerobic sludge against ISfinder database.** (Ranked by sequencing number of the identified insertion sequences in anaerobic sludge).
(DOCX)
