# Supplementary material for: Metagenomic Profiling of Antibiotic Resistance Genes and Mobile Genetic Elements in a Tannery Wastewater Treatment Plant
Source: PLoS One. 2013 Oct 1;8(10):e76079. doi: 10.1371/journal.pone.0076079 (PMC3787945; doi:10.1371/journal.pone.0076079)
Supplement: Figure S1 — Operational processes of the full-scale tannery wastewater treatment plant. A: Influent; B: Adjusting tank; C: Flocculant dosing system; D: Primary sedimentation Tank; E: Up-flow anaerobic sludge reactor; F: Integrated A/O reactor; G: Secondary sedimentation tank; H: Effluent; ①: Influent of up-flow anaerobic sludge reactor; ②: Effluent of up-flow anaerobic sludge reactor; ③: Effluent of integrated A/O reactor. The anaerobic and aerobic sludge samples were collected from Site E and the last aerobic tank of Site F, respectively. The water samples were collected from Sites ①, ② and ③ for water quality analysis. (DOCX) [file pone.0076079.s001.docx]

**Figure S1 Operational processes of the full-scale tannery wastewater treatment plant located in Xiangcheng City (Henan Province, China).**

**
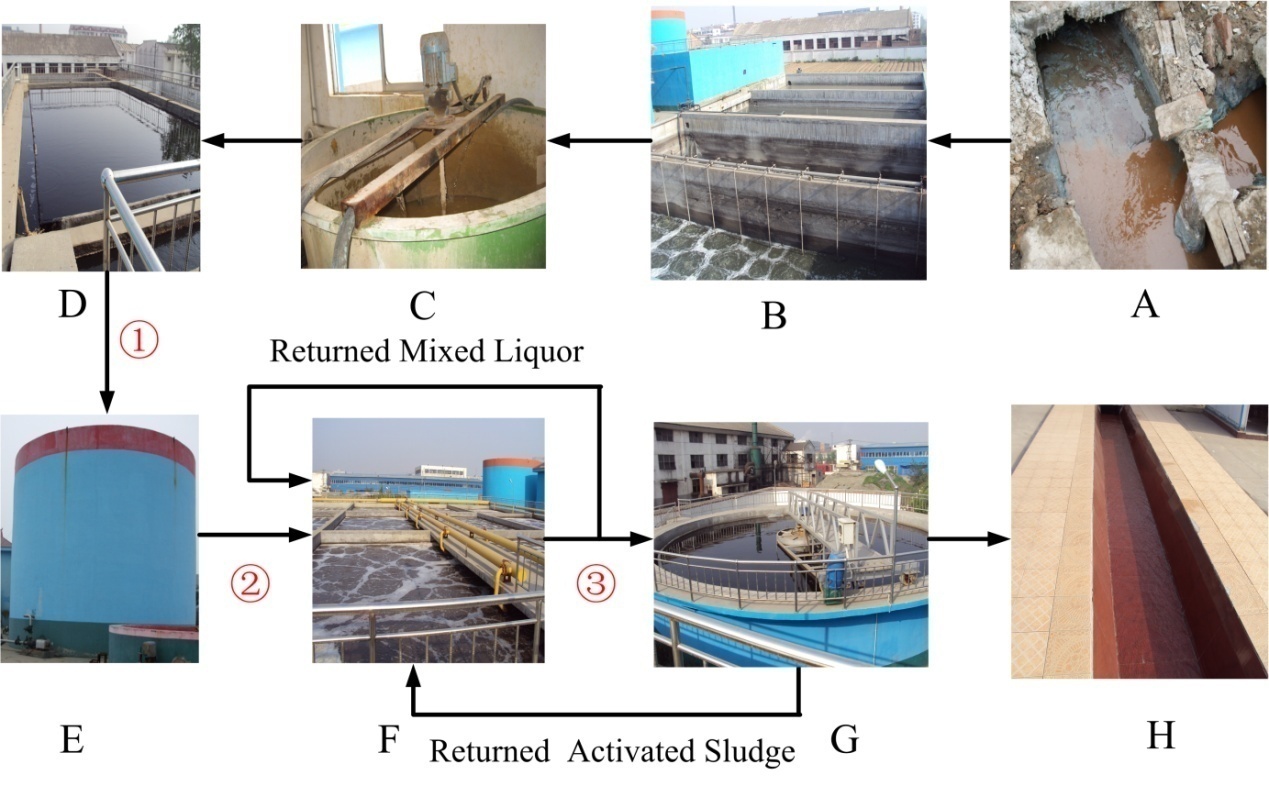
**

A: Influent; B: Adjusting tank; C: Flocculant dosing system; D: Primary sedimentation Tank; E: Up-flow anaerobicsludge reactor; F: Integrated A/O reactor; G: Secondary sedimentation tank; H: Effluent; ①: Influent of up-flow anaerobic sludge reactor; ②: Effluent of up-flow anaerobic sludge reactor; ③: Effluent of integrated A/O reactor. The anaerobic and aerobic sludge samples were collected from Site E and the last aerobic tank of Site F, respectively. The water samples were collected from Sites ①, ② and ③ for water quality analysis.
