# Supplementary material for: Metagenomic Profiling of Antibiotic Resistance Genes and Mobile Genetic Elements in a Tannery Wastewater Treatment Plant
Source: PLoS One. 2013 Oct 1;8(10):e76079. doi: 10.1371/journal.pone.0076079 (PMC3787945; doi:10.1371/journal.pone.0076079)

**Figure S2 Combined taxonomic domain of anaerobic and aerobic sludge.** Each sequencing read is assigned to bacteria, eukaryota, archaea, viruses, and other sequences.


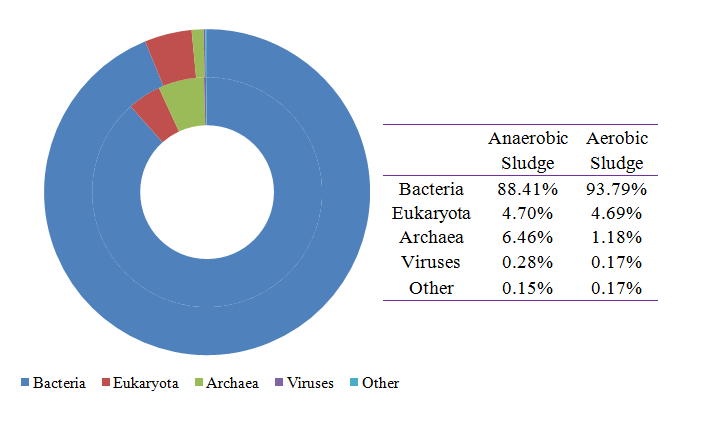

Supplement: Figure S2 — Combined taxonomic domain of anaerobic and aerobic sludge. Each sequencing read is assigned to bacteria, eukaryota, archaea, viruses, and other sequences. (DOCX) [file pone.0076079.s002.docx]
