# Supplementary material for: Metagenomic Profiling of Antibiotic Resistance Genes and Mobile Genetic Elements in a Tannery Wastewater Treatment Plant
Source: PLoS One. 2013 Oct 1;8(10):e76079. doi: 10.1371/journal.pone.0076079 (PMC3787945; doi:10.1371/journal.pone.0076079)

**Figure S3 Functional analysis of the microbial community in anaerobic and aerobic sludge by using MG-RAST annotation.** This figure shows the relative distribution of level 2 categories in level 1 categories of protein metabolism (A), stress response (B), and virulence, disease and defense (C).


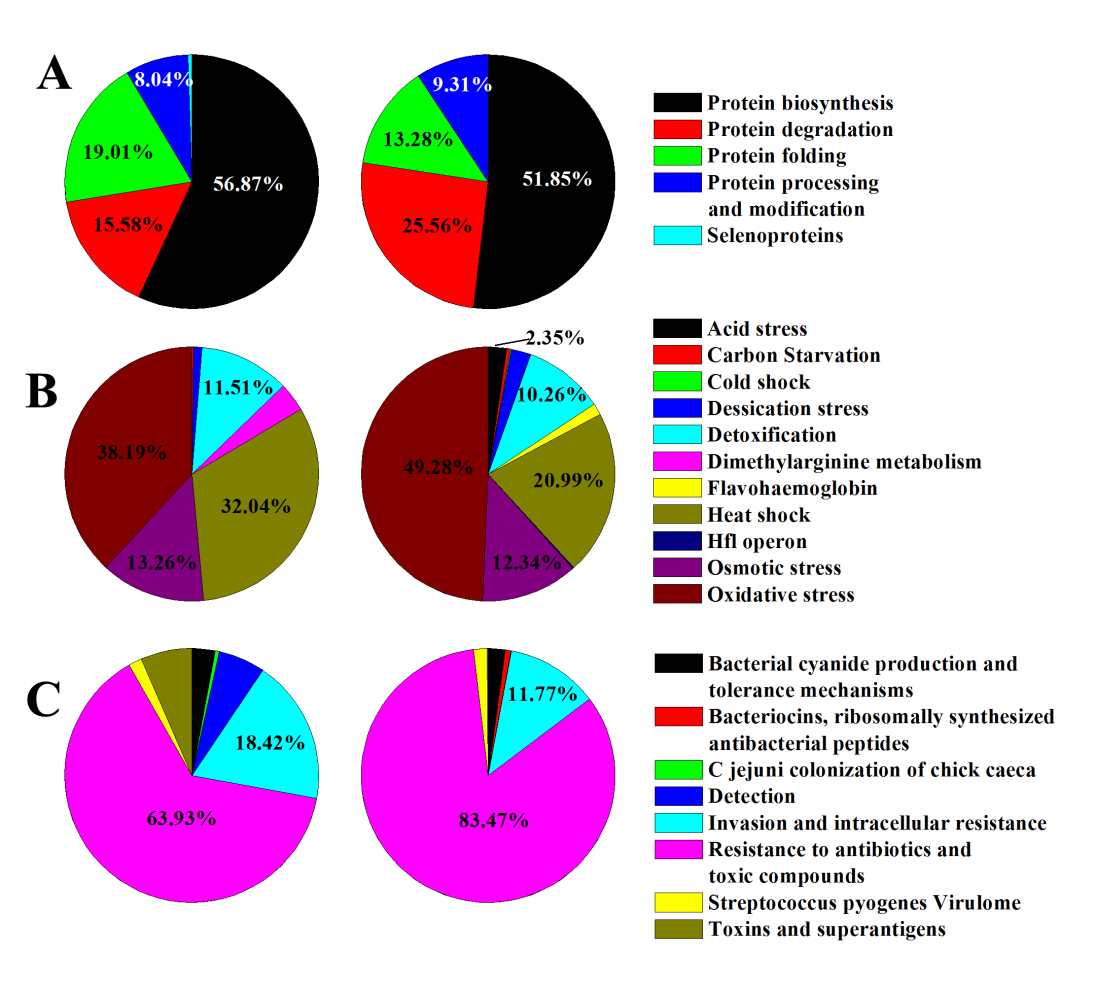

Supplement: Figure S3 — Functional analysis of the microbial community in anaerobic and aerobic sludge by using MG-RAST annotation. This figure shows the relative distribution of level 2 categories in level 1 categories of protein metabolism (A), stress response (B), and virulence, disease and defense (C). (DOCX) [file pone.0076079.s003.docx]
