# Supplementary material for: Metagenomic Profiling of Antibiotic Resistance Genes and Mobile Genetic Elements in a Tannery Wastewater Treatment Plant
Source: PLoS One. 2013 Oct 1;8(10):e76079. doi: 10.1371/journal.pone.0076079 (PMC3787945; doi:10.1371/journal.pone.0076079)
Supplement: Figure S4 — Relative abundance and of different tetracycline resistance genes (tet) in anaerobic and aerobic sludge. (DOCX) [file pone.0076079.s004.docx]

**Figure S4 Relative abundance of different tetracycline resistance genes (*tet*) in anaerobic and aerobic sludge.**

**
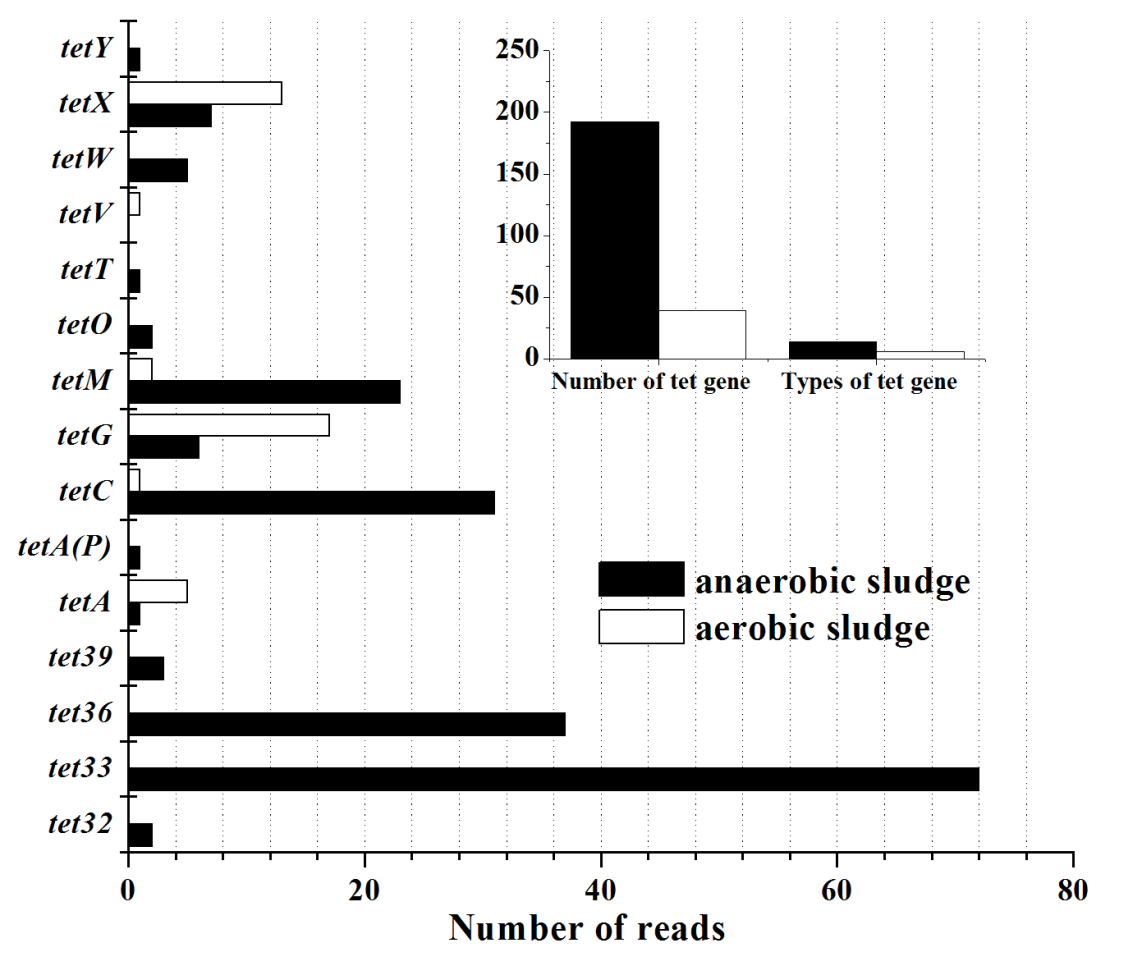
**
