# Supplementary material for: Metagenomic Profiling of Antibiotic Resistance Genes and Mobile Genetic Elements in a Tannery Wastewater Treatment Plant
Source: PLoS One. 2013 Oct 1;8(10):e76079. doi: 10.1371/journal.pone.0076079 (PMC3787945; doi:10.1371/journal.pone.0076079)
Supplement: Table S1 — Operational conditions of the tannery wastewater treatment plants. (DOCX) [file pone.0076079.s005.docx]

**Table S1 Operational conditions of the tannery wastewater treatment plants.**

| Sample | Operational parameters |
| --- | --- |
| Influent of Up-flow anaerobic sludge reactor | COD: 1332 mg/L  Ammonium nitrogen: 177.4 mg/L  Nitrite nitrogen: 0.1 mg/L  Nitrate nitrogen: 2.0 mg/L  Temperature: ~22 ^o^C |
| Effluent of up-flow anaerobic sludge reactor | COD: 610 mg/L  Ammonium nitrogen: 181.6 mg/L  Nitrite nitrogen: 0.04 mg/L  Nitrate nitrogen: 1.3 mg/L  Temperature: ~19 ^o^C |
| Effluent of integrated  A/O reactor | COD: 78 mg/l  Ammonium nitrogen: 5.0 mg/L  Nitrite nitrogen: 0.7 mg/L  Nitrate nitrogen: 77.3 mg/L  Temperature: ~19 ^o^C |
| Up-flow anaerobic sludge reactor | Activated sludge system, no fixed materials (carriers) for bacteria (biofilm) growth  DO: no aeration  pH: 7.5~8.0  Hydraulic retention time: 24 h  MLSS: 10,000~40,000mg/L |
| Integrated A/O reactor | Integrated A/O reactor composed of 12 tanks with equal volume by series connection.  Activated sludge system, no fixed materials (carriers) for bacteria (biofilm) growth  DO: oxic tank (~4mg/L), anoxic tank (<2mg/L)  pH: 7.0~8.0  MLSS: 4,000~5,000mg/L  Hydraulic retention time: 48 h |

COD: chemical oxygen demand; DO: dissolved oxygen; MLSS: [mixed liquor suspended solids.](http://www.norweco.com/html/lab/test_methods/2540dfpmlss.htm)
