# Supplementary material for: Metagenomic Profiling of Antibiotic Resistance Genes and Mobile Genetic Elements in a Tannery Wastewater Treatment Plant
Source: PLoS One. 2013 Oct 1;8(10):e76079. doi: 10.1371/journal.pone.0076079 (PMC3787945; doi:10.1371/journal.pone.0076079)
Supplement: Table S2 — Information of sequence quality control for the metagenomic data of the sludge samples. (DOCX) [file pone.0076079.s006.docx]

**Table S2** **Information of sequence quality control for the metagenomic data of the sludge samples.**

| Sample | anaerobic sludge | | aerobic sludge | |
| --- | --- | --- | --- | --- |
| Total sequences | 11,959,298 | 100.00% | 11,992,160 | 100.00% |
| Filtered by FASTX toolkit | 2,483,901 | 20.77% | 3,276,561 | 27.32% |
| Containing “N” sequences | 3,073 | 0.03% | 2,331 | 0.02% |
| Replicate sequences | 277,391 | 2.32% | 60,948 | 0.51% |
| Passed above QC pipline | 9,194,933 | 76.89% | 8,652,320 | 72.15% |
