# Supplementary material for: Metagenomic Profiling of Antibiotic Resistance Genes and Mobile Genetic Elements in a Tannery Wastewater Treatment Plant
Source: PLoS One. 2013 Oct 1;8(10):e76079. doi: 10.1371/journal.pone.0076079 (PMC3787945; doi:10.1371/journal.pone.0076079)
Supplement: Table S3 — Abundance of different genera in anaerobic and aerobic sludge. The abundance is presented in terms of percentage in total classified sequences in a sample. Representative taxa with abundance of over 0.25% in either anaerobic or aerobic sludge are shown (Sorted alphabetically by domain, phylum and then genus). (DOCX) [file pone.0076079.s007.docx]

**Table S3 Abundance of different genera in anaerobic and aerobic sludge.** The abundance is presented in terms of percentage in total classified sequences in a sample. Representative taxa with abundance of over 0.25% in either anaerobic or aerobic sludge are shown (Sorted alphabetically by domain, phylum and then genus).

| Domain | Phylum | Genus | Percentage | |
| --- | --- | --- | --- | --- |
|  |  |  | anaerobic sludge | aerobic sludge |
| Archaea | *Euryarchaeota* | *Methanococcoides* | 0.25% | 0.02% |
|  |  | *Methanoplanus* | 0.28% | 0.01% |
|  |  | *Methanosaeta* | 1.06% | 0.03% |
|  |  | *Methanosarcina* | 0.93% | 0.13% |
|  |  | *Methanosphaerula* | 0.26% | 0.03% |
|  |  | *Methanospirillum* | 0.30% | 0.03% |
| Bacteria | *Actinobacteria* | *Candidatus Koribacter* | 0.18% | 0.27% |
|  |  | *Candidatus Solibacter* | 0.00% | 0.53% |
|  |  | *Arthrobacter* | 0.19% | 0.26% |
|  |  | *Corynebacterium* | 0.36% | 0.33% |
|  |  | *Frankia* | 0.33% | 0.53% |
|  |  | *Mycobacterium* | 0.62% | 1.06% |
|  |  | *Rhodococcus* | 0.24% | 0.37% |
|  |  | *Streptomyces* | 0.99% | 1.35% |
|  | *α-Proteobacteria* | *Agrobacterium* | 0.11% | 0.45% |
|  |  | *Azospirillum* | 0.09% | 0.25% |
|  |  | *Bradyrhizobium* | 0.32% | 1.23% |
|  |  | *Brucella* | 0.11% | 0.41% |
|  |  | *Caulobacter* | 0.18% | 0.48% |
|  |  | *Chelativorans* | 0.09% | 0.45% |
|  |  | *Erythrobacter* | 0.31% | 0.35% |
|  |  | *Hyphomicrobium* | 0.12% | 0.58% |
|  |  | *Magnetospirillum* | 0.21% | 0.64% |
|  |  | *Mesorhizobium* | 0.15% | 1.01% |
|  |  | *Methylobacterium* | 0.36% | 1.01% |
|  |  | *Nitrobacter* | 0.15% | 1.34% |
|  |  | *Novosphingobium* | 0.26% | 0.30% |
|  |  | *Ochrobactrum* | 0.07% | 0.25% |
|  |  | *Oligotropha* | 0.05% | 0.27% |
|  |  | *Parvibaculum* | 0.10% | 0.32% |
|  |  | *Rhizobium* | 0.33% | 0.99% |
|  |  | *Rhodobacter* | 0.26% | 0.51% |
|  |  | *Rhodopseudomonas* | 0.36% | 1.47% |
|  |  | *Rhodospirillum* | 0.17% | 0.52% |
| Bacteria | *α-Proteobacteria* | *Roseobacter* | 0.18% | 0.34% |
|  |  | *Ruegeria* | 0.22% | 0.32% |
|  |  | *Sinorhizobium* | 0.24% | 0.86% |
|  |  | *Sphingobium* | 0.10% | 0.50% |
|  |  | *Sphingomonas* | 0.17% | 0.74% |
|  |  | *Sphingopyxis* | 0.11% | 0.37% |
|  |  | *Xanthobacter* | 0.10% | 0.38% |
|  | *β-Proteobacteria* | *Methylibium* | 0.09% | 0.36% |
|  |  | *Achromobacter* | 0.14% | 0.36% |
|  |  | *Acidovorax* | 0.62% | 1.27% |
|  |  | *Aromatoleum* | 0.16% | 0.88% |
|  |  | *Azoarcus* | 0.11% | 1.07% |
|  |  | *Bordetella* | 0.23% | 0.65% |
|  |  | *Burkholderia* | 0.95% | 2.21% |
|  |  | *Comamonas* | 0.11% | 0.27% |
|  |  | *Cupriavidus* | 0.24% | 0.85% |
|  |  | *Dechloromonas* | 0.15% | 0.52% |
|  |  | *Delftia* | 0.12% | 0.30% |
|  |  | *Leptothrix* | 0.09% | 0.29% |
|  |  | *Methylobacillus* | 0.09% | 0.25% |
|  |  | *Nitrosomonas* | 0.21% | 1.19% |
|  |  | *Nitrosospira* | 0.10% | 0.46% |
|  |  | *Polaromonas* | 0.32% | 0.76% |
|  |  | *Ralstonia* | 0.19% | 0.59% |
|  |  | *Thauera* | 0.00% | 1.63% |
|  |  | *Thiobacillus* | 0.48% | 0.41% |
|  |  | *Variovorax* | 0.15% | 0.38% |
|  |  | *Verminephrobacter* | 0.14% | 0.31% |
|  | *δ-Proteobacteria* | *Anaeromyxobacter* | 0.37% | 0.48% |
|  |  | *Desulfatibacillum* | 0.41% | 0.10% |
|  |  | *Desulfobacterium* | 0.49% | 0.09% |
|  |  | *Desulfobulbus* | 1.15% | 0.06% |
|  |  | *Desulfococcus* | 0.37% | 0.09% |
|  |  | *Desulfomicrobium* | 1.96% | 0.10% |
|  |  | *Desulfotalea* | 0.46% | 0.08% |
|  |  | *Desulfovibrio* | 1.80% | 0.61% |
|  |  | *Desulfurivibrio* | 0.28% | 0.07% |
|  |  | *Desulfuromonas* | 0.37% | 0.06% |
|  |  | *Geobacter* | 1.32% | 0.84% |
|  |  | *Myxococcus* | 0.19% | 0.48% |
|  |  | *Pelobacter* | 0.55% | 0.29% |
| Bacteria | *δ-Proteobacteria* | *Sorangium* | 0.16% | 0.45% |
|  |  | *Stigmatella* | 0.12% | 0.26% |
|  |  | *Syntrophobacter* | 0.51% | 0.23% |
|  |  | *Syntrophus* | 0.43% | 0.13% |
|  | *γ-Proteobacteria* | *Acinetobacter* | 0.21% | 0.31% |
|  |  | *Allochromatium* | 0.12% | 0.55% |
|  |  | *Escherichia* | 0.25% | 0.28% |
|  |  | *Legionella* | 0.15% | 0.25% |
|  |  | *Marinobacter* | 0.22% | 0.36% |
|  |  | *Methylococcus* | 0.14% | 0.34% |
|  |  | *Nitrosococcus* | 0.25% | 0.60% |
|  |  | *Pseudomonas* | 0.88% | 1.75% |
|  |  | *Shewanella* | 0.57% | 0.66% |
|  |  | *Vibrio* | 0.48% | 0.51% |
|  |  | *Xanthomonas* | 0.55% | 0.61% |
|  | *Bacteroidetes* | *Alistipes* | 0.58% | 0.08% |
|  |  | *Bacteroides* | 3.57% | 0.78% |
|  |  | *Chitinophaga* | 0.41% | 0.43% |
|  |  | *Cytophaga* | 0.33% | 0.30% |
|  |  | *Dyadobacter* | 0.32% | 0.27% |
|  |  | *Flavobacterium* | 0.57% | 0.35% |
|  |  | *Gramella* | 0.35% | 0.23% |
|  |  | *Paludibacter* | 0.40% | 0.06% |
|  |  | *Parabacteroides* | 1.06% | 0.19% |
|  |  | *Pedobacter* | 0.58% | 0.30% |
|  |  | *Porphyromonas* | 0.57% | 0.10% |
|  |  | *Prevotella* | 1.12% | 0.27% |
|  |  | *Sphingobacterium* | 0.25% | 0.15% |
|  |  | *Spirosoma* | 0.35% | 0.31% |
|  | *Chlorobi* | *Chlorobium* | 0.66% | 0.36% |
|  | *Chloroflexi* | *Anaerolinea* | 0.89% | 0.47% |
|  |  | *Chloroflexus* | 0.31% | 0.39% |
|  |  | *Herpetosiphon* | 0.17% | 0.31% |
|  |  | *Roseiflexus* | 0.52% | 0.65% |
|  | *Cyanobacteria* | *Cyanothece* | 0.30% | 0.35% |
|  |  | *Synechococcus* | 0.46% | 0.51% |
|  | *Deinococcus-Thermus* | *Deinococcus* | 0.23% | 0.28% |
|  | *Firmicutes* | *Alkaliphilus* | 0.47% | 0.09% |
|  |  | *Bacillus* | 1.48% | 0.87% |
|  |  | *Caldanaerobacter* | 0.26% | 0.08% |
|  |  | *Caldicellulosiruptor* | 0.29% | 0.09% |
| Bacteria | *Firmicutes* | *Clostridium* | 2.90% | 0.74% |
|  |  | *Desulfitobacterium* | 0.35% | 0.10% |
|  |  | *Desulfotomaculum* | 0.39% | 0.14% |
|  |  | *Enterococcus* | 0.25% | 0.12% |
|  |  | *Eubacterium* | 0.40% | 0.12% |
|  |  | *Geobacillus* | 0.29% | 0.19% |
|  |  | *Lactobacillus* | 0.49% | 0.26% |
|  |  | *Paenibacillus* | 0.46% | 0.27% |
|  |  | *Staphylococcus* | 0.25% | 0.14% |
|  |  | *Streptococcus* | 0.48% | 0.22% |
|  |  | *Syntrophomonas* | 0.25% | 0.06% |
|  |  | *Thermoanaerobacter* | 0.25% | 0.08% |
|  | *Fusobacteria* | *Fusobacterium* | 0.32% | 0.10% |
|  | *Gemmatimonadetes* | *Gemmatimonas* | 0.11% | 0.28% |
|  | *Nitrospirae* | *Nitrospira* | 0.00% | 0.41% |
|  | *Planctomycetes* | *Blastopirellula* | 0.20% | 0.69% |
|  |  | *Gemmata* | 0.13% | 0.41% |
|  |  | *Pirellula* | 0.19% | 0.88% |
|  |  | *Planctomyces* | 0.35% | 1.14% |
|  |  | *Rhodopirellula* | 0.27% | 0.83% |
|  | *Spirochaetes* | *Spirochaeta* | 0.34% | 0.09% |
|  |  | *Treponema* | 0.29% | 0.06% |
|  | *Synergistetes* | *Aminobacterium* | 0.43% | 0.02% |
|  |  | *Dethiosulfovibrio* | 0.33% | 0.03% |
|  |  | *Thermanaerovibrio* | 0.26% | 0.03% |
|  | *Thermotogae* | *Thermotoga* | 0.39% | 0.10% |
|  | *Verrucomicrobia* | *Chthoniobacter* | 0.12% | 0.35% |
|  |  | *Opitutus* | 0.19% | 0.44% |
|  |  | *Candidatus Cloacamonas* | 2.91% | 0.02% |
