# Supplementary material for: Metagenomic Profiling of Antibiotic Resistance Genes and Mobile Genetic Elements in a Tannery Wastewater Treatment Plant
Source: PLoS One. 2013 Oct 1;8(10):e76079. doi: 10.1371/journal.pone.0076079 (PMC3787945; doi:10.1371/journal.pone.0076079)
Supplement: Table S4 — Relative distribution of level 3 categories in level 2 category of “resistance to antibiotics and toxic compounds” based on MG-RASR analysis. (DOCX) [file pone.0076079.s008.docx]

**Table S4** **Relative distribution of level 3 categories in level 2 category of “resistance to antibiotics and toxic compounds” based on MG-RAST analysis.**

| Category | Percentage | |
| --- | --- | --- |
|  | anaerobic  sludge | aerobic  sludge |
| Aminoglycoside adenylyltransferases | 0.84% | 0.54% |
| Arsenic resistance | 9.33% | 12.25% |
| β-lactamase | 2.49% | 0.38% |
| BlaR1 family regulatory sensor-transducer disambiguation | 0.00% | 0.02% |
| Cadmium resistance | 0.02% | 0.00% |
| Cobalt-zinc-cadmium resistance | 4.11% | 2.42% |
| Copper homeostasis | 4.22% | 15.31% |
| Copper homeostasis: copper tolerance | 5.00% | 0.14% |
| Erythromycin resistance | 0.01% | 0.00% |
| Lysozyme inhibitors | 0.43% | 0.00% |
| Mercury resistance operon | 8.55% | 0.64% |
| Methicillin resistance in *Staphylococci* | 34.99% | 35.59% |
| MexA-MexB-OprM multidrug efflux system | 0.12% | 0.15% |
| Polymyxin synthetase gene cluster in *Bacillus* | 0.00% | 1.41% |
| Resistance to chromium compounds | 0.00% | 0.46% |
| Resistance to fluoroquinolones | 29.88% | 30.67% |
| Teicoplanin-resistance in *Staphylococcus* | 0.00% | 0.03% |
