# Supplementary material for: Metagenomic Profiling of Antibiotic Resistance Genes and Mobile Genetic Elements in a Tannery Wastewater Treatment Plant
Source: PLoS One. 2013 Oct 1;8(10):e76079. doi: 10.1371/journal.pone.0076079 (PMC3787945; doi:10.1371/journal.pone.0076079)
Supplement: Table S5 — Matched high-throughput sequencing reads of ARGs in anaerobic and aerobic sludge against ARDB. (Ranked by sequencing number of the identified ARGs in anaerobic sludge). (DOCX) [file pone.0076079.s009.docx]

**Table S5 Matched high-throughput sequencing reads of antibiotic resistance genes (ARGs) in anaerobic and aerobic sludge against Antibiotic Resistance Database (ARDB)** (Ranked by sequencing number of the identified ARGs in anaerobic sludge)

| No | ARDB accession number | ARG name | Identity (%) ≥ | Hit length (AA) ≥ | E value  ≤ | Number of reads | | Function | Related antibiotics |
| --- | --- | --- | --- | --- | --- | --- | --- | --- | --- |
|  |  |  |  |  |  | anaerobic  sludge | aerobic  sludge |  |  |
| 1 | CAE53425 | *sul2* | 96 | 25 | 7.00E-08 | 57 | 79 | Sulfonamide-resistant dihydropteroate synthase | [sulfonamide](http://ardb.cbcb.umd.edu/cgi/search.cgi?db=B&field=ab&term=sulfonamide) |
| 2 | NP_940742 | *tet33* | 96.88 | 26 | 4.00E-10 | 53 | ND | Tetracycline efflux pump | tetracycline |
| 3 | ABG36700 | *sul1* | 90.62 | 31 | 1.00E-10 | 47 | 81 | Sulfonamide-resistant dihydropteroate synthase | sulfonamide |
| 4 | YP_002894485 | *aadA* | 90.62 | 25 | 5.00E-09 | 44 | 46 | Aminoglycoside O-nucleotidylyl transferase | spectinomycin,streptomycin |
| 5 | CAD55718 | *tet36* | 90.62 | 31 | 2.00E-10 | 37 | ND | Ribosomal protection protein | tetracycline |
| 6 | YP_002112964 | *strA* | 96.88 | 25 | 1.00E-09 | 33 | 34 | Aminoglycoside O-phosphotransferase | [streptomycin](http://ardb.cbcb.umd.edu/cgi/search.cgi?db=B&field=ab&term=streptomycin) |
| 7 | ABO42050 | *aadB* | 90.91 | 26 | 3.00E-09 | 32 | 13 | Aminoglycoside O-nucleotidylyltransferase | dibekacin,gentamicin,kanamycin,  sisomicin,tobramycin |
| 8 | YP_002029849 | *smeE* | 90 | 30 | 6.00E-09 | 27 | 26 | Multidrug resistance efflux pump | fluoroquinolone |
| 9 | BAF36555 | *sul1* | 96.88 | 26 | 6.00E-09 | 20 | 44 | Sulfonamide-resistant dihydropteroate synthase | sulfonamide |
| 10 | NP_511233 | *tetC* | 100 | 32 | 2.00E-12 | 20 | 1 | Tetracycline efflux pump | tetracycline |
| 11 | YP_001096238 | *tet33* | 90.62 | 26 | 1.00E-07 | 19 | ND | Tetracycline efflux pump | tetracycline |
| 12 | AAR21617 | *sul1* | 90.62 | 32 | 5.00E-12 | 16 | 29 | Sulfonamide-resistant dihydropteroatesynthase | sulfonamide |
| 13 | ABQ96629 | *sul1* | 96.15 | 25 | 2.00E-08 | 16 | 30 | Sulfonamide-resistant dihydropteroate synthase | sulfonamide |
| 14 | CAC86407 | *strB* | 96.88 | 32 | 4.00E-13 | 12 | 15 | Aminoglycoside O-phosphotransferase | [streptomycin](http://ardb.cbcb.umd.edu/cgi/search.cgi?db=B&field=ab&term=streptomycin) |
| 15 | CAI46978 | *bacA* | 96.88 | 32 | 1.00E-11 | 12 | ND | Class A beta-lactamase | carbenicillin,penicillin |
| 16 | YP_316450 | *bacA* | 90.62 | 31 | 2.00E-10 | 11 | 1 | Undecaprenyl pyrophosphate phosphatase | [bacitracin](http://ardb.cbcb.umd.edu/cgi/search.cgi?db=B&field=ab&term=bacitracin) |
| 17 | CAM88415 | *strB* | 93.55 | 31 | 2.00E-11 | 10 | 8 | Aminoglycoside O-phosphotransferase | [streptomycin](http://ardb.cbcb.umd.edu/cgi/search.cgi?db=B&field=ab&term=streptomycin) |
| 18 | AAK69613 | *ereA* | 92.59 | 27 | 5.00E-09 | 9 | 1 | Erythromycin esterase | erythromycin |
| 19 | CAC47934 | *ermF* | 93.75 | 27 | 4.00E-10 | 9 | 12 | rRNA adenine N-6-methyltransferase | lincosamide,macrolide,streptogramin_b |
| 20 | XP_002333050 | *tetC* | 100 | 32 | 5.00E-12 | 9 | ND | Tetracycline efflux pump | tetracycline |
| 21 | YP_001908403 | *acrB* | 90.32 | 26 | 1.00E-08 | 9 | 6 | Multidrug resistance efflux pump | acriflavin,aminoglycoside, β-lactam,glycylcycline,macrolide |
| 22 | AAO24820 | *tetM* | 100 | 32 | 3.00E-13 | 8 | ND | Ribosomal protection protein | tetracycline |
| 23 | ACR66841 | *sul1* | 96.15 | 26 | 2.00E-10 | 8 | 12 | Sulfonamide-resistant dihydropteroatesynthase | sulfonamide |
| 24 | ACN65403 | *sul1* | 100 | 30 | 8.00E-12 | 7 | 11 | Sulfonamide-resistant dihydropteroate synthase | sulfonamide |
| 25 | YP_001571041 | *macB* | 90 | 29 | 1.00E-09 | 7 | 14 | Multidrug resistance efflux pump | [macrolide](http://ardb.cbcb.umd.edu/cgi/search.cgi?db=B&field=ab&term=macrolide) |
| 26 | YP_002030219 | *smeB* | 90.91 | 31 | 9.00E-11 | 7 | 3 | Multidrug resistance efflux pump | [fluoroquinolone](http://ardb.cbcb.umd.edu/cgi/search.cgi?db=B&field=ab&term=fluoroquinolone) |
| 27 | ZP_04055482 | *ermF* | 93.94 | 32 | 1.00E-11 | 7 | 5 | rRNA adenine N-6-methyltransferase | lincosamide,macrolide,streptogramin_b |
| 28 | ABZ01843 | *tetG* | 90.62 | 32 | 1.00E-11 | 6 | 13 | Tetracycline efflux pump | tetracycline |
| 29 | ACB20261 | *strB* | 100 | 32 | 1.00E-12 | 6 | 13 | Aminoglycoside O-phosphotransferase | [streptomycin](http://ardb.cbcb.umd.edu/cgi/ssquery.cgi?db=T&gn=aph6id) |
| 30 | CAG34265 | *smeE* | 90.62 | 32 | 3.00E-10 | 6 | 5 | Multidrug resistance efflux pump | fluoroquinolone |
| 31 | YP_586061 | *mexB* | 90.62 | 31 | 6.00E-10 | 6 | 20 | Multidrug resistance efflux pump | aminoglycoside, β-lactam,fluoroquinolone, tetracycline,tigecycline |
| 32 | ZP_01974653 | *aadA* | 93.75 | 32 | 1.00E-11 | 6 | 2 | Aminoglycoside O-nucleotidylyltransferase | spectinomycin,streptomycin |
| 33 | AAU89126 | *aacA4* | 90.32 | 31 | 9.00E-11 | 5 | 17 | Aminoglycoside N-acetyltransferase | amikacin,dibekacin,isepamicin,  netilmicin,sisomicin,tobramycin |
| 34 | CAC35725 | *acrB* | 93.75 | 32 | 2.00E-11 | 5 | 1 | Multidrug resistance efflux pump | acriflavin, aminoglycoside, β-lactam, glycylcycline,macrolide |
| 35 | AAL51021 | *aacA4* | 96.88 | 32 | 3.00E-12 | 4 | 6 | Aminoglycoside N-acetyltransferase | amikacin,dibekacin,isepamicin,  netilmicin,sisomicin,tobramycin |
| 36 | AAZ91706 | *ereA* | 96.88 | 32 | 4.00E-12 | 4 | ND | Erythromycin esterase | [erythromycin](http://ardb.cbcb.umd.edu/cgi/search.cgi?db=B&field=ab&term=erythromycin) |
| 37 | ABG81258 | *tetW* | 96.97 | 33 | 3.00E-12 | 4 | ND | Ribosomal protection protein | tetracycline |
| 38 | ABW72062 | *acrB* | 90.91 | 29 | 2.00E-10 | 4 | 2 | Multidrug resistance efflux pump | acriflavin, aminoglycoside, β-lactam, glycylcycline,macrolide |
| 39 | ACI02941 | *tetX* | 100 | 33 | 4.00E-13 | 4 | 2 | NADP-requiring oxidoreductase | tetracycline |
| 40 | BAC77251 | *acrB* | 90.62 | 32 | 1.00E-10 | 4 | 9 | Multidrug resistance efflux pump | acriflavin, aminoglycoside, β-lactam, glycylcycline,macrolide |
| 41 | CAE50480 | *sul1* | 100 | 32 | 4.00E-13 | 4 | 15 | Sulfonamide-resistant dihydropteroate synthase | sulfonamide |
| 42 | Q53770 | *tetM* | 100 | 32 | 1.00E-12 | 4 | 1 | Ribosomal protection protein | tetracycline |
| 43 | YP_001563294 | *bacA* | 90.62 | 32 | 5.00E-11 | 4 | 5 | Undecaprenyl pyrophosphate phosphatase | [bacitracin](http://ardb.cbcb.umd.edu/cgi/search.cgi?db=B&field=ab&term=bacitracin) |
| 44 | YP_001834888 | *tetM* | 100 | 33 | 5.00E-14 | 4 | ND | Ribosomal protection protein | tetracycline |
| 45 | YP_454364 | *acrB* | 90.62 | 30 | 1.00E-09 | 4 | 1 | Multidrug resistance efflux pump | acriflavin, aminoglycoside, β-lactam, glycylcycline,macrolide |
| 46 | ZP_03075977 | *arnA* | 90.32 | 31 | 8.00E-10 | 4 | ND | bifunctional polymyxin resistance protein | [polymyxin](http://ardb.cbcb.umd.edu/cgi/search.cgi?db=B&field=ab&term=polymyxin) |
| 47 | ZP_03835677 | *acrB* | 90.32 | 31 | 1.00E-10 | 4 | 2 | Multidrug resistance efflux pump | acriflavin, aminoglycoside, β-lactam, glycylcycline,macrolide |
| 48 | AAS45561 | *tetM* | 100 | 32 | 1.00E-11 | 3 | ND | Ribosomal protection protein | tetracycline |
| 49 | AAW66497 | *tet39* | 100 | 32 | 1.00E-12 | 3 | ND | Tetracycline efflux pump | tetracycline |
| 50 | ACK77685 | *acrB* | 90.32 | 31 | 9.00E-11 | 3 | 1 | Multidrug resistance efflux pump | acriflavin, aminoglycoside, β-lactam, glycylcycline,macrolide |
| 51 | CAC47932 | *tetX* | 96.97 | 32 | 3.00E-13 | 3 | 9 | NADP-requiring oxidoreductase | tetracycline |
| 52 | YP_010878 | *bacA* | 90.91 | 32 | 2.00E-12 | 3 | 1 | Undecaprenyl pyrophosphate phosphatase | [bacitracin](http://ardb.cbcb.umd.edu/cgi/search.cgi?db=B&field=ab&term=bacitracin) |
| 53 | ZP_04405450 | *strB* | 96.43 | 27 | 6.00E-13 | 3 | 4 | Aminoglycoside O-phosphotransferase | [streptomycin](http://ardb.cbcb.umd.edu/cgi/search.cgi?db=B&field=ab&term=streptomycin) |
| 54 | AAA27431 | *ermF* | 100 | 32 | 3.00E-13 | 2 | 1 | rRNA adenine N-6-methyltransferase | lincosamide,macrolide,streptogramin_b |
| 55 | AAP83161 | *mefA* | 93.75 | 32 | 6.00E-13 | 2 | 1 | Macrolide-Lincosamide-Streptogramin B efflux pump | [macrolide](http://ardb.cbcb.umd.edu/cgi/search.cgi?db=B&field=ab&term=macrolide) |
| 56 | ABV82122 | *tet32* | 100 | 33 | 6.00E-13 | 2 | ND | Ribosomal protection protein | tetracycline |
| 57 | ACI02017 | *tetC* | 100 | 33 | 3.00E-13 | 2 | ND | Tetracycline efflux pump | tetracycline |
| 58 | BAE54319 | *ereB* | 96.97 | 33 | 1.00E-12 | 2 | ND | Erythromycin esterase | [erythromycin](http://ardb.cbcb.umd.edu/cgi/search.cgi?db=B&field=ab&term=erythromycin) |
| 59 | BAH18720 | *ermB* | 96.88 | 32 | 2.00E-12 | 2 | ND | rRNA adenine N-6-methyltransferase | lincosamide,macrolide,streptogramin_b |
| 60 | CAM88409 | *cmlA* | 93.94 | 32 | 1.00E-11 | 2 | 12 | Chloramphenicol efflux pump | [chloramphenicol](http://ardb.cbcb.umd.edu/cgi/search.cgi?db=B&field=ab&term=chloramphenicol) |
| 61 | CAQ56289 | *ermA* | 96.97 | 33 | 2.00E-12 | 2 | ND | rRNA adenine N-6-methyltransferase | lincosamide,macrolide,streptogramin_b |
| 62 | CAY51926 | *mexW* | 90.32 | 31 | 1.00E-09 | 2 | 1 | Multidrug resistance efflux pump |  |
| 63 | EEB58431 | *mexF* | 90.32 | 31 | 3.00E-11 | 2 | 2 | Multidrug resistance efflux pump | chloramphenicol, fluoroquinolone |
| 64 | NP_744962 | *mexB* | 90.62 | 32 | 3.00E-10 | 2 | 1 | Multidrug resistance efflux pump | erythromycin,fluoroquinolone,  glycylcycline,roxithromycin |
| 65 | YP_001716198 | *ermT* | 100 | 32 | 1.00E-12 | 2 | ND | rRNA adenine N-6-methyltransferase | lincosamide,macrolide,streptogramin_b |
| 66 | YP_001836040 | *ermB* | 100 | 32 | 1.00E-12 | 2 | ND | rRNA adenine N-6-methyltransferase | lincosamide,macrolide,streptogramin_b |
| 67 | YP_001969930 | *sul2* | 100 | 27 | 1.00E-09 | 2 | 7 | Sulfonamide-resistant dihydropteroate synthase | [sulfonamide](http://ardb.cbcb.umd.edu/cgi/search.cgi?db=B&field=ab&term=sulfonamide) |
| 68 | YP_002240037 | *acrB* | 96.88 | 31 | 2.00E-11 | 2 | ND | Multidrug resistance efflux pump | acriflavin, aminoglycoside, β-lactam, glycylcycline,macrolide |
| 69 | YP_002384138 | *mdtF* | 90.91 | 33 | 3.00E-10 | 2 | ND | Multidrug resistance efflux pump | doxorubicin,erythromycin |
| 70 | YP_002890644 | *bacA* | 90.62 | 31 | 7.00E-11 | 2 | 63 | Undecaprenyl pyrophosphate phosphatase | [bacitracin](http://ardb.cbcb.umd.edu/cgi/search.cgi?db=B&field=ab&term=bacitracin) |
| 71 | YP_970399 | *bacA* | 90.91 | 33 | 2.00E-12 | 2 | 3 | Undecaprenyl pyrophosphate phosphatase | [bacitracin](http://ardb.cbcb.umd.edu/cgi/search.cgi?db=B&field=ab&term=bacitracin) |
| 72 | YP_985461 | *bacA* | 90 | 30 | 8.00E-10 | 2 | 3 | Undecaprenyl pyrophosphate phosphatase | [bacitracin](http://ardb.cbcb.umd.edu/cgi/search.cgi?db=B&field=ab&term=bacitracin) |
| 73 | ZP_01363330 | *mexB* | 90.32 | 31 | 3.00E-10 | 2 | 11 | Multidrug resistance efflux pump | aminoglycoside,β-lactam,  fluoroquinolone,tetracycline,tigecycline |
| 74 | AAA63165 | *ermF* | 100 | 33 | 8.00E-15 | 1 | ND | rRNA adenine N-6-methyltransferase | Lincosamide,macrolide,streptogramin_b |
| 75 | AAG21808 | *floR* | 96.97 | 32 | 2.00E-12 | 1 | 5 | Chloramphenicol efflux pump | [chloramphenicol](http://ardb.cbcb.umd.edu/cgi/search.cgi?db=B&field=ab&term=chloramphenicol) |
| 76 | AAL05554 | *lnuB* | 100 | 33 | 2.00E-14 | 1 | ND | Lincosamide nucleotidyltransferase | lincomycin |
| 77 | AAN06707 | *tetA* | 100 | 33 | 1.00E-12 | 1 | 2 | Tetracycline efflux pump | tetracycline |
| 78 | AAP22012 | *cmx* | 96.97 | 32 | 2.00E-11 | 1 | 2 | Chloramphenicol efflux pump | chloramphenicol |
| 79 | AAP43641 | *bla_OXA-53_* | 90.62 | 32 | 6.00E-15 | 1 | ND | Class D beta-lactamase | cloxacillin,penicillin |
| 80 | AAQ92181 | *mexB* | 90.32 | 31 | 2.00E-10 | 1 | 19 | Multidrug resistance efflux pump | aminoglycoside, β-lactam, fluoroquinolone,tetracycline,tigecycline |
| 81 | AAV80410 | *tetT* | 100 | 32 | 1.00E-13 | 1 | ND | Ribosomal protection protein | tetracycline |
| 82 | ABG71589 | *mdtF* | 100 | 33 | 9.00E-14 | 1 | ND | Multidrug resistance efflux pump | doxorubicin,erythromycin |
| 83 | ABI81214 | *tetM* | 96.97 | 33 | 9.00E-14 | 1 | ND | Ribosomal protection protein | tetracycline |
| 84 | ABQ41445 | *tetY* | 100 | 33 | 2.00E-14 | 1 | ND | Tetracycline efflux pump | tetracycline |
| 85 | ABR13271 | *acrB* | 93.94 | 33 | 8.00E-12 | 1 | ND | Multidrug resistance efflux pump | acriflavin, aminoglycoside, β-lactam, glycylcycline,macrolide |
| 86 | ABR19665 | *tetM* | 100 | 33 | 7.00E-14 | 1 | ND | Ribosomal protection protein | tetracycline |
| 87 | ABV57476 | *tetM* | 100 | 32 | 3.00E-12 | 1 | 1 | Ribosomal protection protein | tetracycline |
| 88 | ACM50321 | *sul3* | 96 | 25 | 3.00E-08 | 1 | ND | Sulfonamide-resistant dihydropteroate synthase | [sulfonamide](http://ardb.cbcb.umd.edu/cgi/search.cgi?db=B&field=ab&term=sulfonamide) |
| 89 | ACN73425 | *sul1* | 93.94 | 33 | 2.00E-11 | 1 | 1 | Sulfonamide-resistant dihydropteroate synthase | sulfonamide |
| 90 | BAB71966 | *tetA(P)* | 90.62 | 32 | 3.00E-10 | 1 | ND | Tetracycline efflux pump | tetracycline |
| 91 | CAG74079 | [*acrB*](http://ardb.cbcb.umd.edu/cgi/ssquery.cgi?db=T&gn=ceob) | 93.55 | 31 | 9.00E-11 | 1 | 4 | Multidrug resistance efflux pump | acriflavin, aminoglycoside, β-lactam, glycylcycline,macrolide |
| 92 | CAJ77026 | [*strB*](http://ardb.cbcb.umd.edu/cgi/ssquery.cgi?db=T&gn=ceob) | 96.3 | 27 | 3.00E-11 | 1 | 1 | Aminoglycoside O-phosphotransferase | [streptomycin](http://ardb.cbcb.umd.edu/cgi/search.cgi?db=B&field=ab&term=streptomycin) |
| 93 | NP_044444 | *cmr* | 100 | 33 | 2.00E-11 | 1 | 1 | Chloramphenicol efflux pump | [chloramphenicol](http://ardb.cbcb.umd.edu/cgi/search.cgi?db=B&field=ab&term=chloramphenicol) |
| 94 | NP_598114 | *mexB* | 90.62 | 32 | 9.00E-11 | 1 | 6 | Multidrug resistance efflux pump | erythromycin,fluoroquinolone,  glycylcycline,roxithromycin |
| 95 | YP_001038094 | *vatB* | 100 | 33 | 8.00E-15 | 1 | ND | Virginiamycin A acetyltransferase | [streptogramin_a](http://ardb.cbcb.umd.edu/cgi/search.cgi?db=B&field=ab&term=streptogramin_a) |
| 96 | YP_001175677 | *acrB* | 90.91 | 33 | 5.00E-11 | 1 | ND | Multidrug resistance efflux pump | acriflavin, aminoglycoside, β-lactam, glycylcycline,macrolide |
| 97 | YP_001187667 | *bacA* | 92.31 | 26 | 5.00E-08 | 1 | ND | Undecaprenyl pyrophosphate phosphatase | [bacitracin](http://ardb.cbcb.umd.edu/cgi/search.cgi?db=B&field=ab&term=bacitracin) |
| 98 | YP_001187672 | *mexF* | 90.91 | 25 | 7.00E-08 | 1 | 2 | Multidrug resistance efflux pump | chloramphenicol, fluoroquinolone |
| 99 | YP_001350280 | *mexW* | 90 | 30 | 2.00E-09 | 1 | 2 | Multidrug resistance efflux pump |  |
| 100 | YP_001454232 | *acrB* | 96.97 | 33 | 5.00E-12 | 1 | ND | Multidrug resistance efflux pump | acriflavin, aminoglycoside, β-lactam, glycylcycline,macrolide |
| 101 | YP_001477378 | *rosA* | 90.62 | 32 | 1.00E-11 | 1 | 1 | Efflux pump | [fosmidomycin](http://ardb.cbcb.umd.edu/cgi/search.cgi?db=B&field=ab&term=fosmidomycin) |
| 102 | YP_001785579 | *vatB* | 100 | 33 | 6.00E-15 | 1 | ND | Virginiamycin A acetyltransferase | [streptogramin_a](http://ardb.cbcb.umd.edu/cgi/search.cgi?db=B&field=ab&term=streptogramin_a) |
| 103 | YP_002029850 | *smeD* | 90.32 | 31 | 2.00E-09 | 1 | ND | Multidrug resistance efflux pump | [fluoroquinolone](http://ardb.cbcb.umd.edu/cgi/search.cgi?db=B&field=ab&term=fluoroquinolone) |
| 104 | YP_002434803 | *bacA* | 93.94 | 33 | 8.00E-12 | 1 | ND | Undecaprenyl pyrophosphate phosphatase | [bacitracin](http://ardb.cbcb.umd.edu/cgi/search.cgi?db=B&field=ab&term=bacitracin) |
| 105 | YP_251227 | *ermX* | 96.97 | 33 | 4.00E-13 | 1 | ND | rRNA adenine N-6-methyltransferase | lincosamide,macrolide,streptogramin_b |
| 106 | YP_298289 | *mexB* | 90.62 | 32 | 7.00E-11 | 1 | ND | Multidrug resistance efflux pump | aminoglycoside, β-lactam, fluoroquinolone, tetracycline, tigecycline |
| 107 | YP_594556 | *tetW* | 90 | 30 | 8.00E-10 | 1 | ND | Ribosomal protection protein | tetracycline |
| 108 | YP_857635 | *ampC* | 90.91 | 33 | 8.00E-12 | 1 | ND | Class C beta-lactamase | [cephalosporin](http://ardb.cbcb.umd.edu/cgi/search.cgi?db=B&field=ab&term=cephalosporin) |
| 109 | YP_932216 | *bacA* | 93.75 | 32 | 3.00E-11 | 1 | 2 | Undecaprenyl pyrophosphate phosphatase | [bacitracin](http://ardb.cbcb.umd.edu/cgi/search.cgi?db=B&field=ab&term=bacitracin) |
| 110 | ZP_01996651 | *tetO* | 100 | 33 | 3.00E-13 | 1 | ND | Ribosomal protection protein | tetracycline |
| 111 | ZP_02848503 | *vanRB* | 96.88 | 32 | 9.00E-14 | 1 | ND | VanB type vancomycin resistance operon genes | [vancomycin](http://ardb.cbcb.umd.edu/cgi/search.cgi?db=B&field=ab&term=vancomycin) |
| 112 | ZP_03015699 | *ermG* | 100 | 32 | 9.00E-14 | 1 | ND | rRNA adenine N-6-methyltransferase | lincosamide,macrolide,streptogramin_b |
| 113 | ZP_03060937 | *macB* | 90.62 | 32 | 3.00E-10 | 1 | 1 | Multidrug resistance efflux pump | [macrolide](http://ardb.cbcb.umd.edu/cgi/search.cgi?db=B&field=ab&term=macrolide) |
| 114 | ZP_03222019 | *acrB* | 90.91 | 32 | 5.00E-11 | 1 | 2 | Multidrug resistance efflux pump | acriflavin, aminoglycoside, β-lactam, glycylcycline,macrolide |
| 115 | ZP_03223548 | *tetO* | 100 | 32 | 2.00E-12 | 1 | ND | Ribosomal protection protein | tetracycline |
| 116 | ZP_03281410 | *acrB* | 90.91 | 33 | 5.00E-11 | 1 | 2 | Multidrug resistance efflux pump | acriflavin, aminoglycoside, β-lactam, glycylcycline,macrolide |
| 117 | ZP_03466816 | *bacA* | 90.91 | 33 | 3.00E-13 | 1 | ND | Undecaprenyl pyrophosphate phosphatase | [bacitracin](http://ardb.cbcb.umd.edu/cgi/search.cgi?db=B&field=ab&term=bacitracin) |
| 118 | ZP_03541894 | *bacA* | 90.62 | 32 | 4.00E-11 | 1 | 2 | Undecaprenyl pyrophosphate phosphatase | [bacitracin](http://ardb.cbcb.umd.edu/cgi/search.cgi?db=B&field=ab&term=bacitracin) |
| 119 | ZP_03823622 | *acrB* | 93.94 | 32 | 5.00E-11 | 1 | 3 | Multidrug resistance efflux pump | acriflavin, aminoglycoside, β-lactam, glycylcycline,macrolide |
| 120 | ZP_04097279 | *vanRB* | 100 | 33 | 7.00E-13 | 1 | ND | VanB type vancomycin resistance operon genes | [vancomycin](http://ardb.cbcb.umd.edu/cgi/search.cgi?db=B&field=ab&term=vancomycin) |
| 121 | ZP_04309403 | *vanRA* | 96.97 | 33 | 3.00E-14 | 1 | ND | VanA type vancomycin resistance operon genes | teicoplanin,vancomycin |
| 122 | ZP_04435342 | *tetM* | 96.97 | 33 | 1.00E-12 | 1 | ND | Ribosomal protection protein | tetracycline |
| 123 | ZP_04552172 | *ermF* | 90.62 | 32 | 2.00E-12 | 1 | ND | rRNA adenine N-6-methyltransferase | lincosamide,macrolide,streptogramin_b |
| 124 | ZP_04615616 | *acrB* | 93.94 | 33 | 2.00E-11 | 1 | ND | Multidrug resistance efflux pump | acriflavin, aminoglycoside, β-lactam, glycylcycline,macrolide |
| 125 | AAB84282 | *tetV* | 100 | 30 | 1.00E-11 | ND | 1 | Tetracycline efflux pump | tetracycline |
| 126 | AAK14791 | *bla_NPS-1_* | 93.94 | 33 | 7.00E-14 | ND | 1 | Class D beta-lactamase | cloxacillin,penicillin |
| 127 | AAK55330 | *bla_OXA_* | 96.97 | 33 | 3.00E-13 | ND | 2 | Class D beta-lactamase | cloxacillin,penicillin |
| 128 | AAQ21216 | [*acrB*](http://ardb.cbcb.umd.edu/cgi/ssquery.cgi?db=T&gn=acrb) | 90.62 | 32 | 1.00E-10 | ND | 2 | Multidrug resistance efflux pump | acriflavin, aminoglycoside, β-lactam, glycylcycline,macrolide |
| 129 | AAS89471 | [*catB3*](http://ardb.cbcb.umd.edu/cgi/ssquery.cgi?db=T&gn=catb3) | 100 | 32 | 1.00E-12 | ND | 1 | Group B chloramphenicol acetyltransferase | [chloramphenicol](http://ardb.cbcb.umd.edu/cgi/search.cgi?db=B&field=ab&term=chloramphenicol) |
| 130 | ABB92626 | [*catB3*](http://ardb.cbcb.umd.edu/cgi/ssquery.cgi?db=T&gn=catb3) | 100 | 33 | 1.00E-15 | ND | 2 | Group B chloramphenicol acetyltransferase | [chloramphenicol](http://ardb.cbcb.umd.edu/cgi/search.cgi?db=B&field=ab&term=chloramphenicol) |
| 131 | ABE02098 | [*mefA*](http://ardb.cbcb.umd.edu/cgi/ssquery.cgi?db=T&gn=mefa) | 90.62 | 32 | 3.00E-11 | ND | 1 | Macrolide-Lincosamide-Streptogramin B efflux pump | [macrolide](http://ardb.cbcb.umd.edu/cgi/search.cgi?db=B&field=ab&term=macrolide) |
| 132 | ABM94007 | *bacA* | 100 | 32 | 2.00E-12 | ND | 1 | Undecaprenyl pyrophosphate phosphatase | [bacitracin](http://ardb.cbcb.umd.edu/cgi/search.cgi?db=B&field=ab&term=bacitracin) |
| 133 | ABO47746 | *bla_IMP-18_* | 100 | 32 | 5.00E-15 | ND | 1 | Class B beta-lactamase | carbapenem,cephalosporin,cephamycin,penicillin |
| 134 | ABS19074 | *tetA* | 100 | 33 | 1.00E-13 | ND | 1 | Tetracycline efflux pump | tetracycline |
| 135 | ACA48663 | *aadB* | 92 | 25 | 1.00E-08 | ND | 1 | Aminoglycoside O-nucleotidylyltransferase | dibekacin,gentamicin,kanamycin,sisomicin,tobramycin |
| 136 | ACJ39691 | *sul1* | 96.88 | 32 | 6.00E-13 | ND | 2 | Sulfonamide-resistant dihydropteroate synthase | sulfonamide |
| 137 | ACL31199 | *bla_IMP-13_* | 100 | 33 | 2.00E-14 | ND | 1 | Class B beta-lactamase | carbapenem,cephalosporin,cephamycin,penicillin |
| 138 | ACO79044 | *bacA* | 93.75 | 32 | 1.00E-12 | ND | 1 | Undecaprenyl pyrophosphate phosphatase | [bacitracin](http://ardb.cbcb.umd.edu/cgi/search.cgi?db=B&field=ab&term=bacitracin) |
| 139 | BAC67151 | *tetG* | 90.91 | 32 | 2.00E-11 | ND | 2 | Tetracycline efflux pump | tetracycline |
| 140 | BAC77725 | *tetX* | 93.94 | 33 | 9.00E-14 | ND | 2 | NADP-requiring oxidoreductase | [tetracycline](http://ardb.cbcb.umd.edu/cgi/search.cgi?db=B&field=ab&term=tetracycline) |
| 141 | CAC41338 | *tetA* | 100 | 32 | 1.00E-12 | ND | 2 | Tetracycline efflux pump | tetracycline |
| 142 | CAD60196 | *aacA-aphD* | 100 | 32 | 3.00E-14 | ND | 2 | Aminoglycoside N-acetyltransferase | amikacin,dibekacin,isepamicin,netilmicin,sisomicin,tobramycin |
| 143 | CAG34249 | *smeB* | 90 | 30 | 6.00E-09 | ND | 1 | Multidrug resistance efflux pump | [fluoroquinolone](http://ardb.cbcb.umd.edu/cgi/search.cgi?db=B&field=ab&term=fluoroquinolone) |
| 144 | CAG34257 | *smeE* | 90.62 | 32 | 1.00E-09 | ND | 1 | Multidrug resistance efflux pump | fluoroquinolone |
| 145 | CAP74085 | [*catB3*](http://ardb.cbcb.umd.edu/cgi/ssquery.cgi?db=T&gn=catb3) | 100 | 27 | 1.00E-10 | ND | 1 | Group B chloramphenicol acetyltransferase | [chloramphenicol](http://ardb.cbcb.umd.edu/cgi/search.cgi?db=B&field=ab&term=chloramphenicol) |
| 146 | EEQ96502 | *bacA* | 90.62 | 32 | 3.00E-11 | ND | 4 | Undecaprenyl pyrophosphate phosphatase | [bacitracin](http://ardb.cbcb.umd.edu/cgi/search.cgi?db=B&field=ab&term=bacitracin) |
| 147 | NP_792892 | *mexF* | 93.75 | 32 | 2.00E-11 | ND | 1 | Multidrug resistance efflux pump | chloramphenicol, fluoroquinolone |
| 148 | P51563 | *tetG* | 96.97 | 33 | 1.00E-11 | ND | 2 | Tetracycline efflux pump | tetracycline |
| 149 | Q00983 | *bla_LCR-1_* | 90.62 | 32 | 3.00E-13 | ND | 1 | Class D beta-lactamase | cloxacillin,penicillin |
| 150 | Q51429 | *bla_OXA-3_* | 100 | 32 | 1.00E-13 | ND | 3 | Class D beta-lactamase | cloxacillin,penicillin |
| 151 | Q82Y49 | *bacA* | 90.91 | 32 | 8.00E-12 | ND | 5 | Undecaprenyl pyrophosphate phosphatase | [bacitracin](http://ardb.cbcb.umd.edu/cgi/search.cgi?db=B&field=ab&term=bacitracin) |
| 152 | YP_001346277 | [*mexI*](http://ardb.cbcb.umd.edu/cgi/ssquery.cgi?db=T&gn=mexi) | 90.62 | 28 | 1.00E-08 | ND | 5 | Multidrug resistance efflux pump |  |
| 153 | YP_001416709 | *bacA* | 90.91 | 33 | 3.00E-11 | ND | 1 | Undecaprenyl pyrophosphate phosphatase | [bacitracin](http://ardb.cbcb.umd.edu/cgi/search.cgi?db=B&field=ab&term=bacitracin) |
| 154 | YP_001438541 | *macB* | 90.62 | 32 | 2.00E-10 | ND | 2 | Multidrug resistance efflux pump | [macrolide](http://ardb.cbcb.umd.edu/cgi/search.cgi?db=B&field=ab&term=macrolide) |
| 155 | YP_001477377 | *rosB* | 90.62 | 32 | 3.00E-10 | ND | 1 | Efflux pump | [fosmidomycin](http://ardb.cbcb.umd.edu/cgi/search.cgi?db=B&field=ab&term=fosmidomycin) |
| 156 | YP_001668663 | *mexB* | 90.91 | 33 | 2.00E-11 | ND | 2 | Multidrug resistance efflux pump | erythromycin,fluoroquinolone,glycylcycline,roxithromycin |
| 157 | YP_001747848 | *mexW* | 96.77 | 31 | 4.00E-11 | ND | 1 | Multidrug resistance efflux pump |  |
| 158 | YP_001749316 | *mexF* | 90.62 | 32 | 5.00E-12 | ND | 1 | Multidrug resistance efflux pump | chloramphenicol, fluoroquinolone |
| 159 | YP_002081505 | *mexF* | 90.62 | 32 | 5.00E-11 | ND | 2 | Multidrug resistance efflux pump | chloramphenicol, fluoroquinolone |
| 160 | YP_002239469 | *macB* | 90.32 | 31 | 2.00E-10 | ND | 1 | Multidrug resistance efflux pump | [macrolide](http://ardb.cbcb.umd.edu/cgi/search.cgi?db=B&field=ab&term=macrolide) |
| 161 | YP_002382193 | *macB* | 96.88 | 32 | 5.00E-12 | ND | 1 | Multidrug resistance efflux pump | [macrolide](http://ardb.cbcb.umd.edu/cgi/search.cgi?db=B&field=ab&term=macrolide) |
| 162 | YP_002552549 | *bacA* | 96.88 | 32 | 1.00E-14 | ND | 1 | Undecaprenyl pyrophosphate phosphatase | [bacitracin](http://ardb.cbcb.umd.edu/cgi/search.cgi?db=B&field=ab&term=bacitracin) |
| 163 | YP_002800512 | *mexF* | 90.32 | 31 | 2.00E-10 | ND | 3 | Multidrug resistance efflux pump | chloramphenicol, fluoroquinolone |
| 164 | YP_151445 | *acrB* | 100 | 33 | 3.00E-12 | ND | 3 | Multidrug resistance efflux pump | acriflavin, aminoglycoside, β-lactam, glycylcycline,macrolide |
| 165 | YP_232872 | [*floR*](http://www.ncbi.nlm.nih.gov/gene/4929479) | 96.97 | 33 | 6.00E-13 | ND | 2 | Chloramphenicol efflux pump | [chloramphenicol](http://ardb.cbcb.umd.edu/cgi/search.cgi?db=B&field=ab&term=chloramphenicol) |
| 166 | YP_274481 | *mexF* | 93.55 | 31 | 3.00E-10 | ND | 3 | Multidrug resistance efflux pump | chloramphenicol, fluoroquinolone |
| 167 | YP_348389 | *mexF* | 93.75 | 32 | 3.00E-12 | ND | 1 | Multidrug resistance efflux pump | chloramphenicol, fluoroquinolone |
| 168 | YP_369007 | *amrB* | 93.33 | 30 | 1.00E-09 | ND | 1 | Multidrug resistance efflux pump | acriflavine,aminoglycoside,macrolide |
| 169 | YP_523088 | *bacA* | 90.91 | 33 | 4.00E-11 | ND | 2 | Undecaprenyl pyrophosphate phosphatase | [bacitracin](http://ardb.cbcb.umd.edu/cgi/search.cgi?db=B&field=ab&term=bacitracin) |
| 170 | YP_550152 | *bacA* | 90.32 | 31 | 1.00E-12 | ND | 1 | Undecaprenyl pyrophosphate phosphatase | [bacitracin](http://ardb.cbcb.umd.edu/cgi/search.cgi?db=B&field=ab&term=bacitracin) |
| 171 | YP_606823 | *mexW* | 90.62 | 32 | 9.00E-11 | ND | 1 | Multidrug resistance efflux pump |  |
| 172 | YP_607927 | *mexF* | 90.62 | 32 | 2.00E-10 | ND | 1 | Multidrug resistance efflux pump | chloramphenicol, fluoroquinolone |
| 173 | YP_608682 | *mexB* | 90.62 | 32 | 2.00E-10 | ND | 1 | Multidrug resistance efflux pump | erythromycin,fluoroquinolone,  glycylcycline,roxithromycin |
| 174 | YP_746446 | *bacA* | 90.62 | 32 | 4.00E-12 | ND | 1 | Undecaprenyl pyrophosphate phosphatase | [bacitracin](http://ardb.cbcb.umd.edu/cgi/search.cgi?db=B&field=ab&term=bacitracin) |
| 175 | YP_776582 | *ceoB* | 93.75 | 32 | 4.00E-11 | ND | 1 | Multidrug resistance efflux pump | [chloramphenicol](http://ardb.cbcb.umd.edu/cgi/search.cgi?db=B&field=ab&term=chloramphenicol) |
| 176 | YP_981592 | *bacA* | 93.94 | 33 | 2.00E-12 | ND | 1 | Undecaprenyl pyrophosphate phosphatase | [bacitracin](http://ardb.cbcb.umd.edu/cgi/search.cgi?db=B&field=ab&term=bacitracin) |
| 177 | YP_997055 | *bacA* | 90.62 | 32 | 7.00E-11 | ND | 1 | Undecaprenyl pyrophosphate phosphatase | [bacitracin](http://ardb.cbcb.umd.edu/cgi/search.cgi?db=B&field=ab&term=bacitracin) |
| 178 | ZP_02374815 | *amrB* | 90.62 | 32 | 3.00E-10 | ND | 1 | Multidrug resistance efflux pump | acriflavine,aminoglycoside,macrolide |
| 179 | ZP_02901439 | *mdtF* | 93.94 | 33 | 2.00E-11 | ND | 1 | Multidrug resistance efflux pump | doxorubicin,erythromycin |
| 180 | ZP_02904980 | *ceoB* | 96.97 | 33 | 1.00E-11 | ND | 1 | Multidrug resistance efflux pump | [chloramphenicol](http://ardb.cbcb.umd.edu/cgi/search.cgi?db=B&field=ab&term=chloramphenicol) |
| 181 | ZP_03552050 | *bacA* | 96.3 | 27 | 3.00E-09 | ND | 2 | Undecaprenyl pyrophosphate phosphatase | [bacitracin](http://ardb.cbcb.umd.edu/cgi/search.cgi?db=B&field=ab&term=bacitracin) |
| 182 | ZP_03583212 | *ceoB* | 93.75 | 32 | 1.00E-11 | ND | 3 | Multidrug resistance efflux pump | [chloramphenicol](http://ardb.cbcb.umd.edu/cgi/search.cgi?db=B&field=ab&term=chloramphenicol) |
| 183 | ZP_03790349 | *ceoB* | 90.62 | 32 | 7.00E-06 | ND | 10 | Multidrug resistance efflux pump | [chloramphenicol](http://ardb.cbcb.umd.edu/cgi/search.cgi?db=B&field=ab&term=chloramphenicol) |
| 184 | ZP_04577926 | *bacA* | 90 | 30 | 6.00E-10 | ND | 3 | Undecaprenylpyrophosphate phosphatase | [bacitracin](http://ardb.cbcb.umd.edu/cgi/search.cgi?db=B&field=ab&term=bacitracin) |
| 185 | ZP_04612532 | *acrB* | 90.32 | 31 | 3.00E-10 | ND | 3 | Multidrug resistance efflux pump | acriflavin, aminoglycoside, β-lactam, glycylcycline,macrolide |
| 186 | ZP_04612558 | *rosA* | 90.62 | 32 | 8.00E-12 | ND | 1 | Efflux pump | [fosmidomycin](http://ardb.cbcb.umd.edu/cgi/search.cgi?db=B&field=ab&term=fosmidomycin) |
| 187 | ZP_04623896 | *rosA* | 90.62 | 32 | 7.00E-11 | ND | 1 | Efflux pump | [fosmidomycin](http://ardb.cbcb.umd.edu/cgi/search.cgi?db=B&field=ab&term=fosmidomycin) |
| 188 | ZP_04625767 | *acrB* | 93.33 | 30 | 8.00E-10 | ND | 1 | Multidrug resistance efflux pump | acriflavin, aminoglycoside, β-lactam, glycylcycline,macrolide |

ND: not detectable
