# Supplementary material for: Metagenomic Profiling of Antibiotic Resistance Genes and Mobile Genetic Elements in a Tannery Wastewater Treatment Plant
Source: PLoS One. 2013 Oct 1;8(10):e76079. doi: 10.1371/journal.pone.0076079 (PMC3787945; doi:10.1371/journal.pone.0076079)
Supplement: Table S6 — Matched high-throughput sequencing reads of integron integrase genes in anaerobic and aerobic sludge against the INTEGRALL database. (Ranked by reads number of the identified integron integrase genes in aerobic sludge). (DOCX) [file pone.0076079.s010.docx]

**Table S6 Matched high-throughput sequencing reads of integron integrase genes in anaerobic and aerobic sludge against the INTEGRALL database** (Ranked by reads number of the identified integron integrase genes in aerobic sludge).

| Microbial species | Accession number | Integrase  Gene | Gene Cassette | Identity (%) ≥ | Hit length (bp) ≥ | E value ≤ | Number of reads | |
| --- | --- | --- | --- | --- | --- | --- | --- | --- |
|  |  |  |  |  |  |  | anaerobic sludge | aerobic  sludge |
| *uncultured* | FM866490 | *intI* | *Unknown* | 98 | 68 | 3.00E-30 | 26 | 64 |
| *uncultured* | FJ663011 | *intI1* | *qacE,IS1067* | 98 | 97 | 9.00E-45 | 19 | 52 |
| *uncultured* | FJ820123 | *intI1* | *qacF,qacE* | 96 | 100 | 2.00E-41 | 8 | 41 |
| *Corynebacterium* | BX248359 | *intI1* | *Unknown* | 94.44 | 50 | 6.00E-17 | 1 | 34 |
| *Pseudomonas* | PSEAADA | *intI1* | *aadA2b,sul1* | 94 | 52 | 2.00E-21 | 8 | 24 |
| *Stenotrophomonas* | AF406792 | *intI1* | *smr1,qacEΔ1* | 90.72 | 50 | 3.00E-15 | 23 | 18 |
| *uncultured* | FJ820145 | *intI1* | *qacG* | 92.93 | 98 | 7.00E-36 | 5 | 15 |
| *Salmonella* | DQ836009 | *intI1* | *aadA2,linG* | 95 | 99 | 3.00E-39 | 6 | 14 |
| *Thauera* | EU327987 | *intI1* | *orf,orf,orf,orf,orf,orf,orf,orf,orf,orf* | 95.05 | 90 | 2.00E-37 | ND | 13 |
| *Thauera* | EU327991 | *intI1* | *orf* | 98.94 | 94 | 4.00E-43 | ND | 8 |
| *uncultured* | EU531490 | *intI1* | *Unknown* | 90.1 | 100 | 2.00E-31 | ND | 7 |
| *Salmonella* | AY524415 | *intI1* | *sul1,qacEΔ1,aadA1,dfrA1* | 93.65 | 56 | 7.00E-21 | 3 | 5 |
| *uncultured* | EU531479 | *intI1* | *Unknown* | 95.96 | 98 | 7.00E-41 | ND | 4 |
| *Vibrio* | GQ214169 | *intI1* | *aadA1,qacEΔ1* | 100 | 100 | 4.00E-48 | 2 | 4 |
| *Pseudomonas* | EU503121 | *intI1* | *bla_OXA-18_,aacA4,bla_OXA-20_,dnaK,IS* | 98.36 | 54 | 2.00E-22 | 5 | 3 |
| *uncultured* | FJ377614 | *intI* | *Unknown* | 92 | 100 | 9.00E-35 | ND | 3 |
| *Nitrosomonas* | AL954747 | *intINeu* | *orf* | 97 | 100 | 4.00E-43 | ND | 2 |
| *uncultured* | DQ282219 | *intI* | *Unknown* | 90.32 | 78 | 2.00E-27 | ND | 2 |
| *uncultured* | FJ820142 | *intI1* | *aadA,qacE* | 100 | 100 | 4.00E-48 | ND | 2 |
| *Desulfurivibrio* | NC_014216 | *intI* | *Unknown* | 90.48 | 53 | 1.00E-14 | ND | 2 |
| *Klebsiella* | AY219651 | *intI3* | *bla_GES-1_,bla_OXA_/aacA4,repC* | 96.3 | 81 | 2.00E-32 | ND | 1 |
| *Pseudomonas* | AY257539 | *intI1* | *sul1,qacEΔ,aadB* | 100 | 58 | 9.00E-25 | ND | 1 |
| *Pseudomonas* | AY775051 | *intI1* | *orf11,aacA4,aacC1,bla_VIM-2_* | 100 | 100 | 4.00E-48 | ND | 1 |
| *Serratia* | D50438 | *Unknown* | *Unknown* | 100 | 100 | 4.00E-48 | ND | 1 |
| *Pseudomonas* | DQ522233 | *intI1* | *aacA7,bla_VIM-2_,dfrB5,aacC5* | 100 | 100 | 4.00E-48 | 1 | 1 |
| *uncultured* | EU531491 | *intI1* | *Unknown* | 100 | 100 | 4.00E-48 | ND | 1 |
| *uncultured* | FJ377591 | *intI* | *Unknown* | 96 | 100 | 2.00E-41 | ND | 1 |
| *uncultured* | FJ377601 | *intI* | *Unknown* | 94.83 | 58 | 9.00E-20 | ND | 1 |
| *Klebsiella* | FJ594766 | *intI1* | *aacA4-CR,bla_OXA-1_,catB3,arr2,qacEΔ1* | 100 | 100 | 4.00E-48 | ND | 1 |
| *uncultured* | FJ820160 | *intI1* | *qacE* | 100 | 100 | 4.00E-48 | ND | 1 |
| *Pseudomonas* | EF207719 | *intI1* | *bla_VIM-2_,aacA4,ereA1,qacEΔ1* | 98.02 | 58 | 9.00E-25 | 23 | ND |

ND: not detectable
