# Supplementary material for: Metagenomic Profiling of Antibiotic Resistance Genes and Mobile Genetic Elements in a Tannery Wastewater Treatment Plant
Source: PLoS One. 2013 Oct 1;8(10):e76079. doi: 10.1371/journal.pone.0076079 (PMC3787945; doi:10.1371/journal.pone.0076079)
Supplement: Table S7 — Matched high-throughput sequencing reads of insertion sequences in anaerobic and aerobic sludge against ISfinder database. (Ranked by sequencing number of the identified insertion sequences in anaerobic sludge). (DOCX) [file pone.0076079.s011.docx]

**Table S7** **Matched high-throughput sequencing reads of insertion sequences in anaerobic and aerobic sludge against ISfinder database** (Ranked by sequencing number of the identified insertion sequences in anaerobic sludge).

| Transposon | Accession Number | Identity (%) ≥ | Hit length (bp) ≥ | E value ≤ | Number of reads | | Bacterial hosts |
| --- | --- | --- | --- | --- | --- | --- | --- |
|  |  |  |  |  | anaerobic sludge | aerobic sludge |  |
| IS*Efa4* | [AY082011](http://www.ebi.ac.uk/cgi-bin/emblfetch?AY082011) | 90 | 52 | 4.00E-18 | 133 | ND | *Enterococcus faecium* |
| IS*Ecp1* | [AJ242809](http://www.ebi.ac.uk/cgi-bin/emblfetch?AJ242809) | 90 | 51 | 2.00E-21 | 129 | 2 | *Escherichia coli* |
| IS*Dde1* | [NC_007519](http://www.ebi.ac.uk/cgi-bin/emblfetch?NC_007519) | 90 | 51 | 2.00E-12 | 99 | 2 | *Desulfovibrio desulfuricans* |
| IS*Vsa3* | [AJ289135](http://www.ebi.ac.uk/cgi-bin/emblfetch?AJ289135) | 98 | 53 | 2.00E-22 | 46 | 106 | *Vibrio salmonicida* |
| IS*Pps1* | [AF 342828](http://www.ebi.ac.uk/cgi-bin/emblfetch?AF%20342828) | 90.36 | 52 | 6.00E-22 | 21 | 49 | *Pseudomonas huttiensis* |
| IS*Ppu12* | [AY128707](http://www.ebi.ac.uk/cgi-bin/emblfetch?AY128707) | 96 | 59 | 7.00E-26 | 13 | 40 | *Pseudomonas putida* |
| IS*As1* | [L27156](http://www.ebi.ac.uk/cgi-bin/emblfetch?L27156) | 93.33 | 90 | 4.00E-33 | 6 | 1 | *Aeromonas salmonicida* |
| IS*D1* | [AF034211](http://www.ebi.ac.uk/cgi-bin/emblfetch?AF034211) | 91 | 100 | 4.00E-33 | 6 | ND | *Desulfovibrio vulgaris* |
| IS*Pst2* | [AJ012352](http://www.ebi.ac.uk/cgi-bin/emblfetch?AJ012352) | 97.09 | 57 | 9.00E-25 | 6 | 28 | *Pseudomonas stutzeri* |
| IS*Aba1* | [AY758396](http://www.ebi.ac.uk/cgi-bin/emblfetch?AY758396) | 98 | 100 | 3.00E-45 | 5 | 2 | *Acinetobacter baumannii* |
| IS*Dar2* | [NC_007298](http://www.ebi.ac.uk/cgi-bin/emblfetch?NC_007298) | 90.2 | 51 | 4.00E-13 | 5 | ND | *Dechloromonas aromatica* |
| IS*Kpn3* | [AJ971342](http://www.ebi.ac.uk/cgi-bin/emblfetch?AJ971342) | 100 | 50 | 7.00E-21 | 5 | ND | *Klebsiella pneumoniae* |
| IS*Mbu3* | Unknown | 90.72 | 87 | 2.00E-31 | 5 | ND | *Methanococcoides burtonii* |
| IS*Bthe1* | [NC_004663](http://www.ebi.ac.uk/cgi-bin/emblfetch?NC_004663) | 90.82 | 60 | 3.00E-19 | 4 | ND | *Bacteroides thetaiotaomicron* |
| IS*Gsu1* | [AE017180](http://www.ebi.ac.uk/cgi-bin/emblfetch?AE017180) | 95.29 | 85 | 1.00E-33 | 4 | ND | *Geobacter sulfurreducens* |
| IS*Mca3* | [NC_002977](http://www.ebi.ac.uk/cgi-bin/emblfetch?NC_002977) | 90 | 61 | 3.00E-20 | 4 | 7 | *Methylococcus capsulatus Bath* |
| IS*Pa14* | Unknown | 94.74 | 80 | 3.00E-34 | 4 | ND | *Pseudomonas aeruginosa* |
| IS*Pst1* | [AJ005663](http://www.ebi.ac.uk/cgi-bin/emblfetch?AJ005663) | 95.88 | 97 | 3.00E-40 | 4 | ND | *Pseudomonas stutzeri* |
| IS*Rso7* | [NC_003295](http://www.ebi.ac.uk/cgi-bin/emblfetch?NC_003295) | 91.09 | 85 | 3.00E-30 | 4 | 3 | *Ralstonia solanacearum* |
| IS*Sm2* | [X84038](http://www.ebi.ac.uk/cgi-bin/emblfetch?X84038) | 90 | 51 | 7.00E-16 | 4 | 70 | *Xanthobacter autotrophicus* |
| IS*Flsp1* | [AJ421424](http://www.ebi.ac.uk/cgi-bin/emblfetch?AJ421424) | 92.73 | 55 | 2.00E-16 | 3 | 1 | *Flavobacterium* sp*.* |
| IS*Mca2* | [NC_002977](http://www.ebi.ac.uk/cgi-bin/emblfetch?NC_002977) | 90 | 63 | 4.00E-23 | 3 | 7 | *Methylococcus capsulatus Bath* |
| IS*Tesp3* | [AP008980](http://www.ebi.ac.uk/cgi-bin/emblfetch?AP008980) | 90.82 | 58 | 2.00E-17 | 3 | 9 | *Terrabacter* sp. |
| IS*Aav2* | [AF086815](http://www.ebi.ac.uk/cgi-bin/emblfetch?AF086815) | 100 | 100 | 1.00E-48 | 2 | ND | *Acidovorax avenae* |
| IS*Afe7* | Unknown | 91.04 | 67 | 1.00E-19 | 2 | 3 | *Acidithiobacillus ferrooxidans* |
| IS*Cgl1* | [U85507](http://www.ebi.ac.uk/cgi-bin/emblfetch?U85507) | 97 | 95 | 2.00E-42 | 2 | 3 | *Corynebacterium glutamicum* |
| IS*Efa5* | [AY495588](http://www.ebi.ac.uk/cgi-bin/emblfetch?AY495588) | 100 | 100 | 1.00E-48 | 2 | ND | *Enterococcus faecium* |
| IS*H22* | [NC_002607](http://www.ebi.ac.uk/cgi-bin/emblfetch?NC_002607) | 90.82 | 98 | 2.00E-32 | 2 | ND | *Halobacterium* sp. |
| IS*Hma4* | [NC_006392](http://www.ebi.ac.uk/cgi-bin/emblfetch?NC_006392) | 91 | 95 | 1.00E-33 | 2 | ND | *Haloarcula marismortui* |
| IS*Hne2* | [NC_008358](http://www.ebi.ac.uk/cgi-bin/emblfetch?NC_008358) | 94 | 97 | 1.00E-38 | 2 | 1 | *Hyphomonas neptunium* |
| IS*L1* | [X02734](http://www.ebi.ac.uk/cgi-bin/emblfetch?X02734) | 94 | 50 | 7.00E-16 | 2 | ND | *Lactobacillus casei* |
| IS*Mma14* | [NC_003901](http://www.ebi.ac.uk/cgi-bin/emblfetch?NC_003901) | 100 | 100 | 1.00E-48 | 2 | ND | *Methanosarcina mazei* |
| IS*Mma15* | [NC_003901](http://www.ebi.ac.uk/cgi-bin/emblfetch?NC_003901) | 100 | 100 | 1.00E-48 | 2 | ND | *Methanosarcina mazei* |
| IS*Mma5* | [NC_003901](http://www.ebi.ac.uk/cgi-bin/emblfetch?NC_003901) | 100 | 100 | 1.00E-48 | 2 | ND | *Methanosarcina mazei* |
| IS*Mma7* | [NC_003901](http://www.ebi.ac.uk/cgi-bin/emblfetch?NC_003901) | 93.75 | 96 | 2.00E-36 | 2 | ND | *Methanosarcina mazei* |
| IS*Neu3* | [NC_004757](http://www.ebi.ac.uk/cgi-bin/emblfetch?NC_004757) | 96 | 97 | 6.00E-42 | 2 | 11 | *Nitrosomonas europaea* |
| IS*NGR8* | [AE000091](http://www.ebi.ac.uk/cgi-bin/emblfetch?AE000091) | 90 | 51 | 4.00E-13 | 2 | 4 | *Rhizobium* sp |
| IS*Our1* | [AY177427](http://www.ebi.ac.uk/cgi-bin/emblfetch?AY177427) | 100 | 86 | 7.00E-41 | 2 | ND | *Oligella urethralis* |
| IS*Pfl2* | [AY048765](http://www.ebi.ac.uk/cgi-bin/emblfetch?AY048765) | 92.86 | 87 | 3.00E-35 | 2 | 2 | *Pseudomonas fluorescens* |
| IS*Psp3* | [AB047327](http://www.ebi.ac.uk/cgi-bin/emblfetch?AB047327) | 90.57 | 52 | 4.00E-13 | 2 | 8 | *Pseudomonas* sp. |
| IS*Aba125* | [AY751533](http://www.ebi.ac.uk/cgi-bin/emblfetch?AY751533) | 100 | 99 | 4.00E-48 | 1 | ND | *Acinetobacter baumannii* |
| IS*Azo6* | [NC_006513](http://www.ebi.ac.uk/cgi-bin/emblfetch?NC_006513) | 90.72 | 60 | 2.00E-17 | 1 | 8 | *Azoarcus* sp. |
| IS*Bf4* | [NC_006347](http://www.ebi.ac.uk/cgi-bin/emblfetch?NC_006347) | 92.63 | 95 | 4.00E-33 | 1 | ND | *Bacteroides fragilis* |
| IS*Bm3* | [AF047478](http://www.ebi.ac.uk/cgi-bin/emblfetch?AF047478) | 91.53 | 59 | 2.00E-17 | 1 | ND | *Brucella melitensis* |
| IS*Cbt4* | [NC_007581](http://www.ebi.ac.uk/cgi-bin/emblfetch?NC_007581) | 92.08 | 101 | 3.00E-35 | 1 | ND | *Clostridium botulinum* |
| IS*Cc3* | [NC_002696](http://www.ebi.ac.uk/cgi-bin/emblfetch?NC_002696) | 90.1 | 65 | 2.00E-22 | 1 | 11 | *Caulobacter crescentus* |
| IS*Ec1* | [L02370](http://www.ebi.ac.uk/cgi-bin/emblfetch?L02370) | 99 | 100 | 6.00E-47 | 1 | ND | *Escherichia coli* |
| IS*Ec5* | [L02372](http://www.ebi.ac.uk/cgi-bin/emblfetch?L02372) | 99 | 100 | 6.00E-47 | 1 | ND | *Escherichia coli* |
| IS*H1-8* | [X00805](http://www.ebi.ac.uk/cgi-bin/emblfetch?X00805) | 96.63 | 89 | 2.00E-37 | 1 | ND | *Halobacterium salinarum* |
| IS*H27-2* | [X54433](http://www.ebi.ac.uk/cgi-bin/emblfetch?X54433) | 96.04 | 101 | 6.00E-42 | 1 | ND | *Halobacterium salinarum* |
| IS*H27-3* | [X54434](http://www.ebi.ac.uk/cgi-bin/emblfetch?X54434) | 94.95 | 99 | 9.00E-40 | 1 | ND | *Halobacterium salinarum* |
| IS*H28* | [X59158](http://www.ebi.ac.uk/cgi-bin/emblfetch?U42220,%20X59158) | 93 | 100 | 6.00E-37 | 1 | ND | *Halobacterium salinarum* |
| IS*H29* | [NC_002608](http://www.ebi.ac.uk/cgi-bin/emblfetch?NC_002608) | 98.21 | 56 | 2.00E-22 | 1 | ND | *Halobacterium* sp. |
| IS*H3B* | [AF016485](http://www.ebi.ac.uk/cgi-bin/emblfetch?AF016485) | 94 | 100 | 1.00E-38 | 1 | ND | *Halobacterium salinarum* |
| IS*H8B* | [AF016485](http://www.ebi.ac.uk/cgi-bin/emblfetch?AF016485) | 100 | 63 | 4.00E-28 | 1 | ND | *Halobacterium* sp. |
| IS*Hma1* | [NC_005125](http://www.ebi.ac.uk/cgi-bin/emblfetch?NC_005125) | 94.85 | 97 | 4.00E-38 | 1 | ND | *Haloarcula marismortui* |
| IS*Hne4* | [NC_008358](http://www.ebi.ac.uk/cgi-bin/emblfetch?NC_008358) | 98.96 | 96 | 9.00E-45 | 1 | ND | *Hyphomonas neptunium* |
| IS*Mac16* | [NC_003552](http://www.ebi.ac.uk/cgi-bin/emblfetch?NC_003552) | 91.92 | 99 | 9.00E-35 | 1 | ND | *Methanosarcina acetivorans* |
| IS*Mac4* | [NC_003552](http://www.ebi.ac.uk/cgi-bin/emblfetch?NC_003552) | 96.91 | 97 | 6.00E-42 | 1 | ND | *Methanosarcina acetivorans* |
| IS*Mac8* | [NC_003552](http://www.ebi.ac.uk/cgi-bin/emblfetch?NC_003552) | 96 | 100 | 6.00E-42 | 1 | ND | *Methanosarcina acetivorans* |
| IS*Mba2* | [NC_007355](http://www.ebi.ac.uk/cgi-bin/emblfetch?NC_007355) | 91.75 | 97 | 1.00E-33 | 1 | ND | *Methanosarcina barkeri fusaro* |
| IS*Mma9* | [NC_003901](http://www.ebi.ac.uk/cgi-bin/emblfetch?NC_003901) | 100 | 100 | 1.00E-48 | 1 | ND | *Methanosarcina mazei* |
| IS*Ppa2* | [AY179508](http://www.ebi.ac.uk/cgi-bin/emblfetch?AY179508) | 90.91 | 63 | 2.00E-21 | 1 | 3 | *Paracoccus pantotrophus* |
| IS*Ppa4* | [AY177682](http://www.ebi.ac.uk/cgi-bin/emblfetch?AY177682) | 92.22 | 90 | 7.00E-31 | 1 | ND | *Paracoccus pantotrophus* |
| IS*Ppa5* | [AY225410](http://www.ebi.ac.uk/cgi-bin/emblfetch?AY225410) | 90.72 | 97 | 6.00E-32 | 1 | ND | *Paracoccus pantotrophus* |
| IS*Psy2* | [AB063175](http://www.ebi.ac.uk/cgi-bin/emblfetch?AB063175) | 90.38 | 52 | 1.00E-13 | 1 | ND | *Pseudomonas syringae* pv. |
| IS*Rm14* | [AF134706](http://www.ebi.ac.uk/cgi-bin/emblfetch?AF134706) | 90.1 | 94 | 6.00E-32 | 1 | 21 | *Sinorhizobium meliloti* |
| IS*Rm4-1* | [Y13432](http://www.ebi.ac.uk/cgi-bin/emblfetch?Y13432) | 94.37 | 54 | 4.00E-18 | 1 | 6 | *Rhizobium meliloti* |
| IS*S1N* | [M37395](http://www.ebi.ac.uk/cgi-bin/emblfetch?M37395) | 98.97 | 97 | 3.00E-45 | 1 | ND | *Lactococcus lactis* subsp. |
| IS*Sod9* | [NC_004349](http://www.ebi.ac.uk/cgi-bin/emblfetch?NC_004349) | 90 | 57 | 7.00E-16 | 1 | 16 | *Shewanella oneidensis* |
| IS*Sp1* | [AB021963](http://www.ebi.ac.uk/cgi-bin/emblfetch?AB021963) | 93.68 | 88 | 7.00E-36 | 1 | 37 | *Sphingomonas paucimobilis* |
| IS*Syth1* | [NC_006177](http://www.ebi.ac.uk/cgi-bin/emblfetch?NC_006177) | 92.45 | 53 | 7.00E-16 | 1 | ND | *Symbiobacterium thermophilum* |
| IS*Tesp2* | [AB084235](http://www.ebi.ac.uk/cgi-bin/emblfetch?AB084235) | 92.77 | 83 | 3.00E-29 | 1 | ND | *Terrabacter* sp. |
| IS*Xac2* | [NC_003919](http://www.ebi.ac.uk/cgi-bin/emblfetch?NC_003919) | 92.47 | 93 | 6.00E-32 | 1 | ND | *Xanthomonas axonopodis* pv |
| IS*Xac3* | [NC_003919](http://www.ebi.ac.uk/cgi-bin/emblfetch?NC_003919) | 94.34 | 53 | 2.00E-17 | 1 | ND | *Xanthomonas axonopodis* pv |
| IS*Xax1* | [AY935340](http://www.ebi.ac.uk/cgi-bin/emblfetch?AY935340) | 90.53 | 86 | 7.00E-31 | 1 | 4 | *Xanthomonas axonopodis* pv |
| IS*Aav1* | [AF086815](http://www.ebi.ac.uk/cgi-bin/emblfetch?AF086815) | 91.8 | 60 | 2.00E-17 | ND | 29 | *Acidovorax avenae* |
| IS*Aav3* | [AF086815](http://www.ebi.ac.uk/cgi-bin/emblfetch?AF086815) | 90.91 | 86 | 3.00E-29 | ND | 30 | *Acidovorax avenae* |
| IS*Aba5* | Unknown | 92.86 | 98 | 7.00E-36 | ND | 1 | *Acinetobacter baumannii* |
| IS*Ar1* | K03313 | 96 | 100 | 6.00E-42 | ND | 1 | *Agrobacterium rhizogenes* |
| IS*Azo1* | [NC_006513](http://www.ebi.ac.uk/cgi-bin/emblfetch?NC_006513) | 90 | 78 | 9.00E-25 | ND | 13 | *Azoarcus* sp. |
| IS*Azo2* | [NC_006824](http://www.ebi.ac.uk/cgi-bin/emblfetch?NC_006824) | 94.64 | 56 | 3.00E-19 | ND | 1 | *Azoarcus* sp. |
| IS*Azo4* | [NC_006513](http://www.ebi.ac.uk/cgi-bin/emblfetch?NC_006513) | 90 | 78 | 3.00E-29 | ND | 21 | *Azoarcus* sp. |
| IS*Bcen18* | Unknown | 90.8 | 87 | 2.00E-27 | ND | 2 | *Burkholderia cenocepacia* |
| IS*Blo4* | [NC_004307](http://www.ebi.ac.uk/cgi-bin/emblfetch?NC_004307) | 91.03 | 78 | 3.00E-24 | ND | 2 | *Bifidobacterium longum* |
| IS*Bm2* | [AF047478](http://www.ebi.ac.uk/cgi-bin/emblfetch?AF047478) | 90.12 | 81 | 3.00E-24 | ND | 2 | *Brucella melitensis* |
| IS*Brsp1* | [AF284858](http://www.ebi.ac.uk/cgi-bin/emblfetch?AF284858) | 91.84 | 59 | 7.00E-21 | ND | 2 | *Bradyrhizobium* sp. |
| IS*Bugl1* | [AB011023](http://www.ebi.ac.uk/cgi-bin/emblfetch?AB011023) | 90.28 | 72 | 2.00E-21 | ND | 1 | *Burkholderia glumae* |
| IS*Bvi1* | Unknown | 95 | 68 | 7.00E-31 | ND | 8 | *Burkholderia vietnamiensis* |
| IS*Hne1* | [NC_008358](http://www.ebi.ac.uk/cgi-bin/emblfetch?NC_008358) | 95.59 | 68 | 7.00E-26 | ND | 4 | *Hyphomonas neptunium* |
| IS*Mca5* | [NC_002977](http://www.ebi.ac.uk/cgi-bin/emblfetch?NC_002977) | 90.1 | 96 | 6.00E-32 | ND | 3 | *Methylococcus capsulatus Bath* |
| IS*Mca7* | [NC_002977](http://www.ebi.ac.uk/cgi-bin/emblfetch?NC_002977) | 92.75 | 69 | 2.00E-22 | ND | 1 | *Methylococcus capsulatus Bath* |
| IS*Mlo3* | [AP003008](http://www.ebi.ac.uk/cgi-bin/emblfetch?AP003008) | 97.14 | 70 | 1.00E-28 | ND | 1 | *Mesorhizobium loti* |
| IS*Mmg2* | Unknown | 93.55 | 93 | 3.00E-34 | ND | 1 | *Magnetospirillum magnetotacticum* |
| IS*Neu1* | [NC_004757](http://www.ebi.ac.uk/cgi-bin/emblfetch?NC_004757) | 99 | 100 | 6.00E-47 | ND | 3 | *Nitrosomonas europaea* |
| IS*Posp1* | [NC_007948](http://www.ebi.ac.uk/cgi-bin/emblfetch?NC_007948) | 90.2 | 78 | 2.00E-27 | ND | 10 | *Polaromonas* sp. |
| IS*Ppa3* | [AY177681](http://www.ebi.ac.uk/cgi-bin/emblfetch?AY177681) | 99 | 100 | 6.00E-47 | ND | 2 | *Paracoccus pantotrophus* |
| IS*Ppu16* | [NC_002947](http://www.ebi.ac.uk/cgi-bin/emblfetch?NC_002947) | 91.23 | 57 | 2.00E-16 | ND | 1 | *Pseudomonas putida* |
| IS*Ppu18* | Unknown | 100 | 66 | 9.00E-30 | ND | 1 | *Pseudomonas putida* |
| IS*Ppu7* | [AF292393](http://www.ebi.ac.uk/cgi-bin/emblfetch?AF292393) | 94 | 50 | 7.00E-16 | ND | 1 | *Pseudomonas putida* |
| IS*Psp1* | [M57500](http://www.ebi.ac.uk/cgi-bin/emblfetch?M57500) | 90.91 | 55 | 1.00E-14 | ND | 1 | *Pseudomonas* sp. |
| IS*Psp2* | [M57500](http://www.ebi.ac.uk/cgi-bin/emblfetch?M57500) | 98 | 53 | 2.00E-22 | ND | 14 | *Pseudomonas* sp. |
| IS*Pst3* | [AB088753](http://www.ebi.ac.uk/cgi-bin/emblfetch?AB088753) | 90.72 | 57 | 7.00E-16 | ND | 4 | *Pseudomonas stutzeri* |
| IS*Pst4* | [AY894751](http://www.ebi.ac.uk/cgi-bin/emblfetch?AY894751) | 90.14 | 51 | 2.00E-16 | ND | 2 | *Pseudomonas stutzeri* |
| IS*Psy20* | [NC_005773](http://www.ebi.ac.uk/cgi-bin/emblfetch?NC_005773) | 91.46 | 63 | 2.00E-21 | ND | 2 | *Pseudomonas syringae pv.* |
| IS*R1* | [X06616](http://www.ebi.ac.uk/cgi-bin/emblfetch?X06616) | 92.08 | 56 | 2.00E-17 | ND | 4 | *Rhizobium lupini* |
| IS*Rj1* | [X02581](http://www.ebi.ac.uk/cgi-bin/emblfetch?X02581) | 92.78 | 96 | 3.00E-35 | ND | 9 | *Bradyrhizobium japonicum* |
| IS*Rm12* | [AF087641](http://www.ebi.ac.uk/cgi-bin/emblfetch?AF087641) | 91 | 100 | 1.00E-33 | ND | 1 | *Sinorhizobium meliloti* |
| IS*Rm6* | [X95567](http://www.ebi.ac.uk/cgi-bin/emblfetch?X95567) | 93 | 50 | 2.00E-17 | ND | 3 | *Rhizobium meliloti* |
| IS*Rme4* | [NC_007973](http://www.ebi.ac.uk/cgi-bin/emblfetch?NC_007973) | 90.38 | 51 | 4.00E-13 | ND | 4 | *Cupriavidus metallidurans* |
| IS*Rso10* | [NC_003295](http://www.ebi.ac.uk/cgi-bin/emblfetch?%20NC_003295) | 90.41 | 73 | 2.00E-21 | ND | 1 | *Ralstonia solanacearum* |
| IS*Rso16* | [NC_003295](http://www.ebi.ac.uk/cgi-bin/emblfetch?NC_003295) | 94.74 | 57 | 1.00E-19 | ND | 1 | *Ralstonia solanacearum* |
| IS*Rso5* | [NC_003295](http://www.ebi.ac.uk/cgi-bin/emblfetch?NC_003295) | 91.23 | 57 | 7.00E-16 | ND | 1 | *Ralstonia solanacearum* |
| IS*Rso8* | [NC_003295](http://www.ebi.ac.uk/cgi-bin/emblfetch?NC_003295) | 90.48 | 84 | 7.00E-26 | ND | 1 | *Ralstonia solanacearum* |
| IS*Rsp1* | [U00090](http://www.ebi.ac.uk/cgi-bin/emblfetch?U00090) | 93 | 100 | 6.00E-37 | ND | 1 | *Rhizobium* sp. |
| IS*Rtr1* | [AF041379](http://www.ebi.ac.uk/cgi-bin/emblfetch?AF041379) | 91.01 | 60 | 4.00E-18 | ND | 7 | *Rhizobium tropici* |
| IS*S1W* | [M37396](http://www.ebi.ac.uk/cgi-bin/emblfetch?M37395) | 100 | 100 | 1.00E-48 | ND | 1 | *Lactococcus lactis subsp.* |
| IS*Shsp1* | [AB091693](http://www.ebi.ac.uk/cgi-bin/emblfetch?AB091693) | 94.95 | 99 | 3.00E-39 | ND | 2 | *Sphingomonas* sp. |
| IS*Spo7* | [NC_003911](http://www.ebi.ac.uk/cgi-bin/emblfetch?NC_003911) | 91.01 | 89 | 3.00E-29 | ND | 2 | *Silicibacter pomeroyi* |
| IS*Spo9* | [NC_006569](http://www.ebi.ac.uk/cgi-bin/emblfetch?NC_006569) | 90.72 | 94 | 6.00E-32 | ND | 3 | *Silicibacter pomeroyi* |
| IS*Tesp1* | [AB004563](http://www.ebi.ac.uk/cgi-bin/emblfetch?AB004563) | 91.21 | 66 | 2.00E-21 | ND | 2 | *Terrabacter* sp. |
| IS*UnCu2* | [AY294226](http://www.ebi.ac.uk/cgi-bin/emblfetch?AY294226) | 91.04 | 67 | 1.00E-19 | ND | 1 | Uncultured bacterium |
| IS*UnCu4* | [NC_008055](http://www.ebi.ac.uk/cgi-bin/emblfetch?NC_008055) | 100 | 98 | 2.00E-47 | ND | 1 | Plasmid QKH54 |
| IS*Vsp4* | Unknown | 94.23 | 52 | 2.00E-16 | ND | 1 | *Verrucomicrobium spinosum* |
| IS*Xc5* | [Z73593](http://www.ebi.ac.uk/cgi-bin/emblfetch?Z73593) | 95 | 95 | 3.00E-39 | ND | 3 | *Xanthomonas campestris* pv. |
| IS*Xcd1* | [AF263433](http://www.ebi.ac.uk/cgi-bin/emblfetch?AF263433) | 90.62 | 64 | 1.00E-18 | ND | 1 | *Xanthomonas axonopodis* pv. |
| IS*Xo1* | [AF225214](http://www.ebi.ac.uk/cgi-bin/emblfetch?AF225214) | 94.74 | 57 | 1.00E-19 | ND | 3 | *Xanthomonas oryzae* pv. |
| IS*Xo7* | [AY035401](http://www.ebi.ac.uk/cgi-bin/emblfetch?AY035401) | 91 | 89 | 7.00E-31 | ND | 5 | *Xanthomonas oryzae* pv. |

ND: not detectable
